# Supplementary material for: Haze Exposure Changes the Skin Fungal Community and Promotes the Growth of Talaromyces Strains
Source: Microbiol Spectr. 2022 Dec 12;11(3):e01188-22. doi: 10.1128/spectrum.01188-22 (PMC10269824; doi:10.1128/spectrum.01188-22)
Supplement: Supplemental file 1 — Supplemental material. Download spectrum.01188-22-s0001.pdf, PDF file, 2.5 MB [file spectrum.01188-22-s0001.pdf]

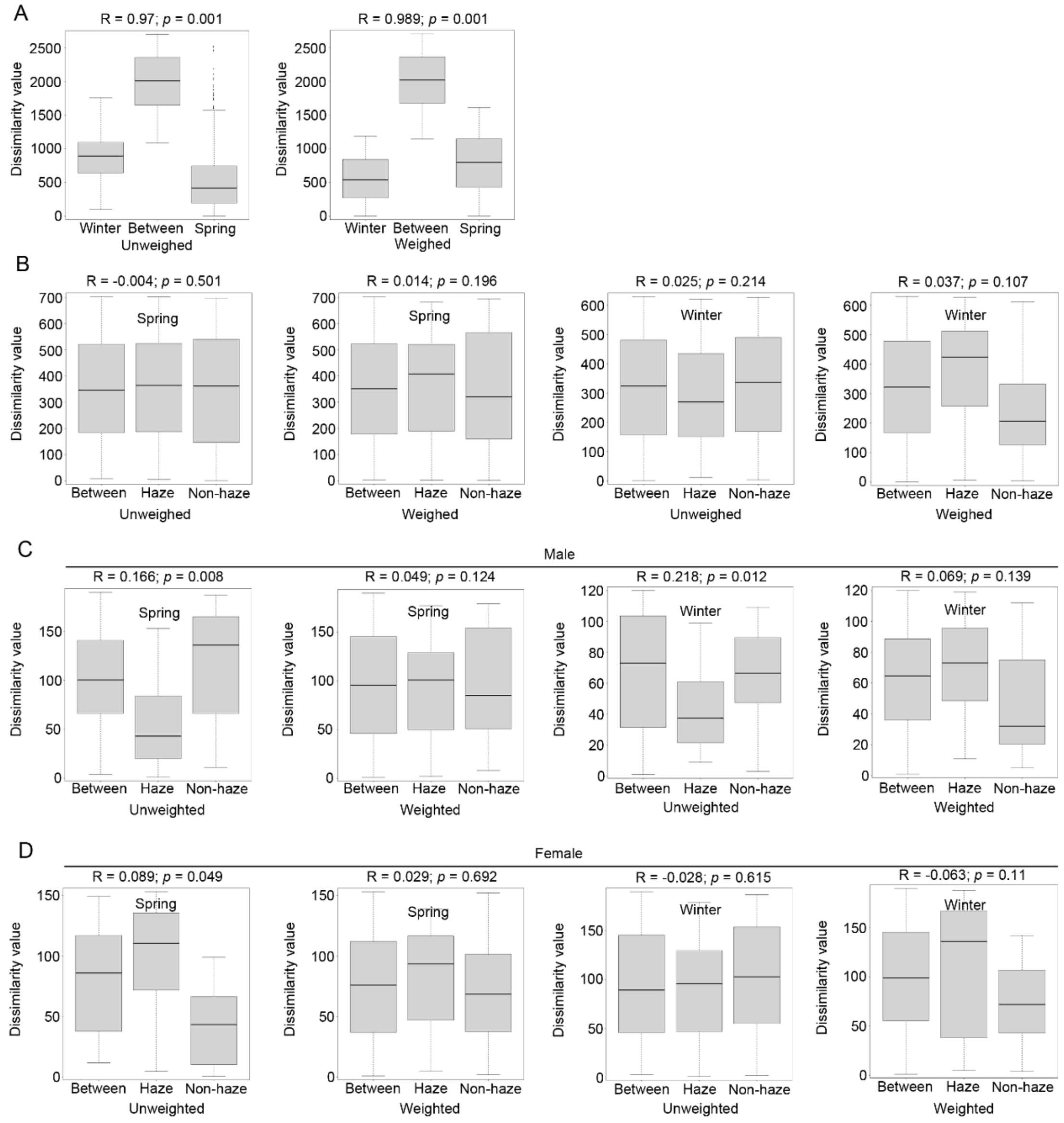


**Fig. S1 Analysis of similarities (ANOSIM) in skin fungal microbiota.** (**A**) ANOSIM indicating the significant changes of skin fungal community between spring and winter; (**B**) ANOSIM indicating no significant changes of skin fungal community were obeserved between haze and non-haze days in spring and winter; (**C**) ANOSIM indicating significant changes of skin fungal community from males between haze and non-haze days; (**D**) ANOSIM indicating significant changes of skin fungal community from males between haze and non-haze days in spring and winter.

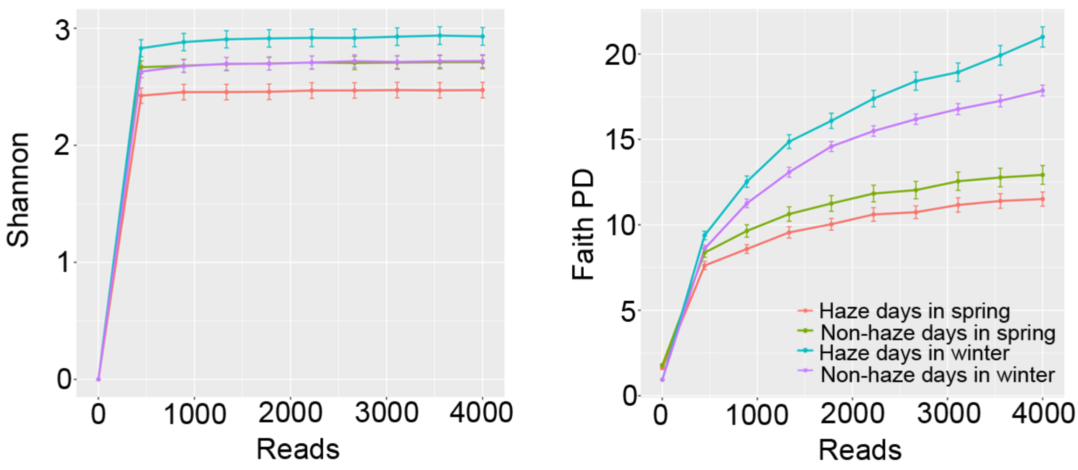


**Fig. S2 Rarefaction curves in all samples based on Shannon and Faith PD index.**

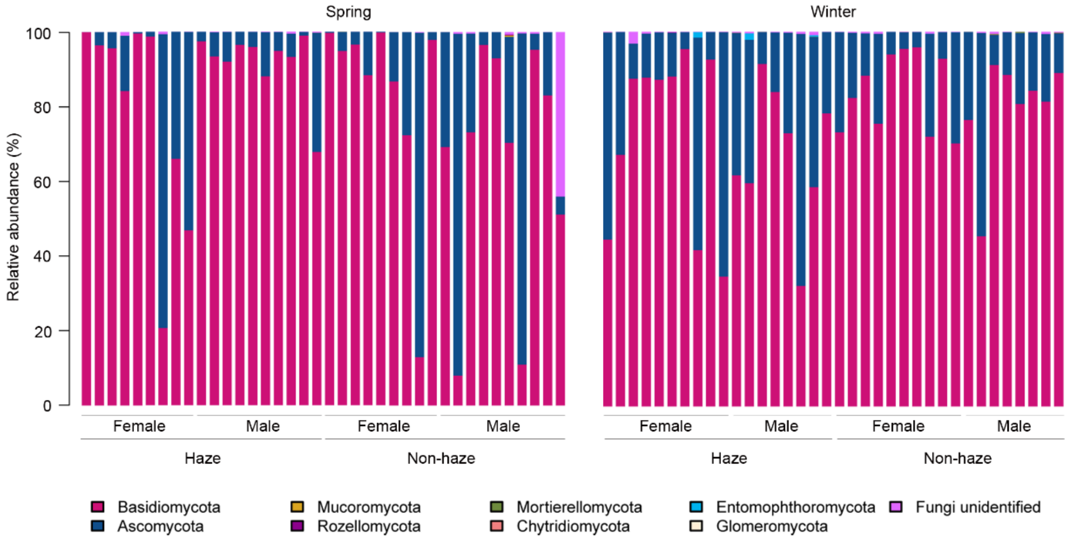


**Fig. S3 Bar plot indicating the relative abundances of dominant phyla**.

**Table S1 The air environmental variables in each sample day**

| Sampling date | AQI | PM2.5 | PM10 | Group |
|---|---|---|---|---|
| 2018/3/8 | 103 | 76 | 118 | haze day in spring |
| 2018/3/9 | 123 | 92 | 144 | haze day in spring |
| 2018/3/10 | 196 | 152 | 215 | haze day in spring |
| 2018/3/11 | 227 | 176 | 247 | haze day in spring |
| 2018/3/12 | 206 | 163 | 232 | haze day in spring |
| 2018/3/13 | 119 | 88 | 140 | haze day in spring |
| 2018/3/14 | 95 | 69 | 108 | haze day in spring |
| Average | 152.7143 | 116.5714 | 172 | |
| 2018/4/21 | 59 | 36 | 64 | non-haze day in spring |
| 2018/4/22 | 41 | 23 | 36 | non-haze day in spring |
| 2018/4/23 | 55 | 37 | 55 | non-haze day in spring |
| 2018/4/24 | 87 | 64 | 95 | non-haze day in spring |
| 2018/4/25 | 108 | 81 | 122 | non-haze day in spring |
| 2018/4/26 | 82 | 54 | 93 | non-haze day in spring |
| 2018/4/27 | 117 | 81 | 145 | non-haze day in spring |
| Average | 78.42857 | 53.71429 | 87.14286 | |
| 2018/11/23 | 122 | 92 | 151 | haze day in winter |
| 2018/11/24 | 205 | 156 | 228 | haze day in winter |
| 2018/11/25 | 239 | 188 | 276 | haze day in winter |
| 2018/11/26 | 217 | 161 | 267 | haze day in winter |
| 2018/11/27 | 235 | 114 | 365 | haze day in winter |
| 2018/11/28 | 293 | 140 | 415 | haze day in winter |
| 2018/11/29 | 168 | 87 | 283 | haze day in winter |
| Average | 211.2857 | 134 | 283.5714 | |
| 2018/12/24 | 84 | 117 | 26 | non-haze day in winter |
| 2018/12/25 | 118 | 165 | 33 | non-haze day in winter |
| 2018/12/26 | 75 | 100 | 14 | non-haze day in winter |
| 2018/12/27 | 78 | 95 | 9 | non-haze day in winter |
| 2018/12/28 | 62 | 74 | 15 | non-haze day in winter |
| 2018/12/29 | 78 | 99 | 15 | non-haze day in winter |
| 2018/12/30 | 84 | 101 | 17 | non-haze day in winter |

**Table S2 The information of samples, factors, and sequences**

| SampleID | haze type | gender | season | pH | water conte | oil content | raw | filtered | denoised | merged | non-chimeric |
|---|---|---|---|---|---|---|---|---|---|---|---|
| A1 | haze | female | Spring | 6.5 | 35 | 37 | 38802 | 26358 | 26281 | 24472 | 24020 |
| A6 | haze | female | Spring | 7 | 36 | 35 | 55640 | 40549 | 40510 | 38123 | 38020 |
| A7 | haze | female | Spring | 6.5 | 36 | 34 | 49174 | 34926 | 34907 | 34439 | 34073 |
| A8 | haze | female | Spring | 7 | 46 | 23 | 36492 | 34867 | 34652 | 31245 | 30634 |
| A13 | haze | female | Spring | 6.5 | 33 | 40 | 55986 | 40930 | 40910 | 40866 | 39572 |
| A17 | haze | female | Spring | 7 | 34 | 38 | 47940 | 34591 | 34570 | 34545 | 33644 |
| A18 | haze | female | Spring | 7 | 33 | 41 | 42934 | 32189 | 32141 | 31322 | 29998 |
| A21 | haze | female | Spring | 7 | 35 | 37 | 69593 | 48500 | 48393 | 45529 | 45524 |
| A23 | haze | female | Spring | 6.3 | 33 | 40 | 71602 | 55528 | 55444 | 55320 | 55230 |
| A26 | haze | male | Spring | 6.5 | 33 | 40 | 72548 | 52925 | 52898 | 51911 | 51911 |
| A28 | haze | male | Spring | 7 | 58 | 18 | 65577 | 62701 | 62435 | 56759 | 55912 |
| A29 | haze | male | Spring | 7 | 21 | 36 | 46189 | 33235 | 33180 | 32983 | 32955 |
| A30 | haze | male | Spring | 6.5 | 29 | 45 | 28001 | 20833 | 20782 | 20710 | 20710 |
| A34 | haze | male | Spring | 6.5 | 43 | 26 | 69755 | 48753 | 48688 | 48485 | 48485 |
| A38 | haze | male | Spring | 6 | 33 | 40 | 41858 | 28186 | 28145 | 27899 | 16425 |
| A41 | haze | male | Spring | 6.5 | 46 | 22 | 36474 | 24030 | 23996 | 23174 | 23160 |
| A46 | haze | male | Spring | 7.5 | 33 | 40 | 51129 | 34744 | 34705 | 34434 | 34408 |
| A48 | haze | male | Spring | 6.5 | 34 | 39 | 35134 | 24250 | 24224 | 23860 | 23578 |
| A50 | haze | male | Spring | 6.5 | 36 | 34 | 41335 | 27199 | 27138 | 25522 | 25507 |
| B1 | non-haze | female | Spring | 6.5 | 35 | 37 | 62041 | 59524 | 59464 | 53369 | 52433 |
| B6 | non-haze | female | Spring | 6.6 | 34 | 38 | 43124 | 32462 | 32394 | 31757 | 29271 |
| B7 | non-haze | female | Spring | 6.7 | 33 | 40 | 73752 | 55540 | 55497 | 55444 | 55444 |
| B8 | non-haze | female | Spring | 5.7 | 36 | 35 | 22207 | 15965 | 15922 | 14376 | 14373 |
| B13 | non-haze | female | Spring | 7 | 46 | 23 | 47643 | 34353 | 34312 | 33844 | 33844 |
| B17 | non-haze | female | Spring | 6.5 | 34 | 39 | 71985 | 53015 | 52923 | 52823 | 52818 |
| B18 | non-haze | female | Spring | 6.7 | 34 | 39 | 41128 | 30132 | 30096 | 30005 | 29965 |
| B21 | non-haze | female | Spring | 6.3 | 43 | 25 | 30878 | 22735 | 22654 | 22095 | 22060 |
| B23 | non-haze | female | Spring | 6.6 | 41 | 27 | 48802 | 33582 | 33505 | 31341 | 31167 |
| B26 | non-haze | male | Spring | 6.5 | 47 | 21 | 39827 | 27815 | 27786 | 26812 | 26504 |
| B28 | non-haze | male | Spring | 6 | 45 | 24 | 74807 | 54236 | 54205 | 53870 | 53137 |
| B29 | non-haze | male | Spring | 6.5 | 20 | 30 | 69704 | 47802 | 47791 | 47073 | 45615 |
| B30 | non-haze | male | Spring | | 28 | 42 | 41127 | 28592 | 28548 | 28504 | 28504 |
| B34 | non-haze | male | Spring | 7 | 25 | 38 | 55199 | 37339 | 37250 | 36647 | 36647 |
| B38 | non-haze | male | Spring | 7 | 25 | 39 | 49883 | 33213 | 33127 | 31695 | 31275 |

| | | | | | | | | | | | |
|---|---|---|---|---|---|---|---|---|---|---|---|
| B41 | non-haze | male | Spring | 7 | 26 | 41 | 71756 | 53695 | 53634 | 53229 | 53202 |
| B46 | non-haze | male | Spring | 6.5 | 21 | 37 | 31405 | 21181 | 21152 | 20637 | 20616 |
| B48 | non-haze | male | Spring | 7 | 29 | 45 | 35997 | 24705 | 24669 | 24258 | 24172 |
| B50 | non-haze | male | Spring | 7 | 33 | 40 | 70492 | 47258 | 47205 | 46761 | 46465 |
| C1 | haze | female | Winter | 6 | 31 | 45 | 39577 | 38791 | 38631 | 38028 | 37840 |
| C4 | haze | female | Winter | 6 | 34 | 39 | 46952 | 45985 | 45918 | 45546 | 45331 |
| C5 | haze | female | Winter | 6.5 | 30 | 46 | 54583 | 53368 | 53326 | 53144 | 53022 |
| C6 | haze | female | Winter | 6 | 24 | 39 | 73849 | 71890 | 71813 | 70793 | 69548 |
| C7 | haze | female | Winter | 6 | 31 | 45 | 68305 | 66501 | 66448 | 66174 | 65391 |
| C9 | haze | female | Winter | 6 | 26 | 41 | 61801 | 60359 | 60285 | 59955 | 59607 |
| C11 | haze | female | Winter | 5.5 | 21 | 36 | 51642 | 50270 | 50249 | 49300 | 49151 |
| C16 | haze | female | Winter | 6 | 24 | 40 | 59603 | 58615 | 58359 | 57635 | 57137 |
| C18 | haze | female | Winter | 6.3 | 31 | 45 | 71324 | 69633 | 69497 | 69303 | 68914 |
| C21 | haze | female | Winter | 6 | 32 | 42 | 63099 | 61234 | 61090 | 60736 | 60413 |
| C26 | haze | male | Winter | 6.5 | 23 | 35 | 62623 | 61028 | 60741 | 59443 | 58571 |
| C27 | haze | male | Winter | 6.5 | 33 | 40 | 56447 | 55432 | 55176 | 54659 | 53138 |
| C31 | haze | male | Winter | 6.8 | 36 | 35 | 62242 | 60705 | 60504 | 56023 | 54715 |
| C33 | haze | male | Winter | 6.5 | 38 | 32 | 71223 | 69337 | 69199 | 68355 | 67122 |
| C35 | haze | male | Winter | 7 | 23 | 41 | 55477 | 53982 | 53706 | 51196 | 49629 |
| C36 | haze | male | Winter | 7 | 21 | 37 | 61608 | 59481 | 59260 | 58771 | 58441 |
| C44 | haze | male | Winter | 7 | 22 | 36 | 71218 | 69529 | 69302 | 68615 | 67705 |
| C46 | haze | male | Winter | 7 | 21 | 37 | 73715 | 72199 | 72142 | 71896 | 71648 |
| D1 | non-haze | female | Winter | 7 | 29 | 45 | 41493 | 40297 | 40233 | 39509 | 39185 |
| D4 | non-haze | female | Winter | 6 | 33 | 40 | 42550 | 41375 | 41173 | 40530 | 40060 |
| D5 | non-haze | female | Winter | 6.5 | 28 | 43 | 42782 | 40360 | 40244 | 39342 | 39137 |
| D6 | non-haze | female | Winter | 6.8 | 28 | 43 | 72505 | 70756 | 70676 | 69368 | 67915 |
| D7 | non-haze | female | Winter | 7 | 20 | 38 | 64934 | 62712 | 62639 | 62361 | 62058 |
| D9 | non-haze | female | Winter | 6.5 | 33 | 40 | 68028 | 66294 | 66265 | 66174 | 65994 |
| D11 | non-haze | female | Winter | 6.5 | 23 | 35 | 62887 | 61186 | 61089 | 58078 | 57444 |
| D16 | non-haze | female | Winter | 6.7 | 29 | 45 | 52406 | 51292 | 51017 | 48974 | 47900 |
| D18 | non-haze | female | Winter | 7 | 47 | 22 | 74133 | 72374 | 72302 | 71911 | 71524 |
| D21 | non-haze | female | Winter | 6.5 | 33 | 44 | 47184 | 46028 | 45856 | 45241 | 44794 |
| D26 | non-haze | male | Winter | 6.8 | 24 | 40 | 63856 | 62310 | 62204 | 60400 | 59899 |
| D27 | non-haze | male | Winter | 6 | 34 | 48 | 62786 | 61466 | 61351 | 60771 | 60678 |
| D31 | non-haze | male | Winter | 6.8 | 17 | 35 | 31356 | 30519 | 30417 | 27249 | 27207 |
| D33 | non-haze | male | Winter | 7 | 23 | 34 | 67833 | 66320 | 66258 | 64287 | 63948 |
| D35 | non-haze | male | Winter | 7 | 23 | 35 | 70257 | 68237 | 68111 | 66649 | 65659 |

| | | | | | | | | | | | |
|---|---|---|---|---|---|---|---|---|---|---|---|
| D36 | non-haze | male | Winter | 7 | 24 | 40 | 55523 | 54017 | 53920 | 52615 | 52399 |
| D44 | non-haze | male | Winter | 7 | 23 | 41 | 69293 | 67307 | 67224 | 66219 | 65101 |
| D46 | non-haze | male | Winter | 6.5 | 20 | 32 | 51034 | 49840 | 49809 | 47684 | 47530 |

**Table S3 The permutation tests between fungal community and interference factors in spring**

| | CCA1 | CCA2 | $r^2$ | *p* |
|---|---|---|---|---|
| gender | -0.59015 | 0.80729 | 0.268 | 0.013* |
| pH | 0.69292 | 0.72101 | 0.0307 | 0.622 |
| water content | -0.90897 | -0.41685 | 0.0156 | 0.8 |
| oil content | 0.99271 | 0.12053 | 0.0938 | 0.232 |

*, $p < 0.05$; **, $p < 0.01$; ***, $p < 0.001$

**Table S4 The permutation tests between fungal community and interference factors in winter**

| | CCA1 | CCA2 | $r^2$ | *p* | |
|---|---|---|---|---|---|
| gender | -0.99522 | -0.09768 | 0.3623 | 0.001*** | |
| pH | -0.48893 | -0.87232 | 0.133 | 0.093 | . |
| water conte | -0.997 | -0.07746 | 0.0566 | 0.394 | |
| oil content | -0.85429 | -0.51979 | 0.0286 | 0.633 | |

*, $p < 0.05$; **, $p < 0.01$; ***, $p < 0.001$

**Table S5 The diversity indices of skin samples**

| Sample ID | Observed_feature | Shannon | PD_whole_tree |
|---|---|---|---|
| A1 | 9 | 2.309738 | 3.804468453 |
| A13 | 16 | 1.948532 | 7.463853403 |
| A17 | 14 | 1.460925 | 4.471492686 |
| A18 | 92 | 4.603396 | 20.01095218 |
| A21 | 40 | 3.291038 | 10.32989403 |
| A23 | 47 | 2.426422 | 14.10962368 |
| A26 | 28 | 1.477881 | 8.121400199 |
| A28 | 83 | 3.699438 | 19.15879639 |
| A29 | 32 | 2.114844 | 10.29216174 |
| A30 | 28 | 1.43801 | 10.25753507 |
| A34 | 57 | 1.58121 | 14.73859755 |
| A38 | 42 | 2.231407 | 11.81714358 |
| A41 | 48 | 2.29137 | 13.28688188 |
| A46 | 64 | 2.189659 | 16.86231375 |
| A48 | 32 | 2.193752 | 11.50669434 |
| A50 | 68 | 4.176553 | 17.54650207 |
| A6 | 29 | 2.946561 | 9.667482584 |
| A7 | 21 | 1.493965 | 8.627141649 |
| A8 | 60 | 3.202276 | 40.50816811 |
| B1 | 17 | 2.22301 | 5.92847339 |
| B13 | 12 | 2.174376 | 5.872876941 |
| B17 | 49 | 2.098308 | 13.56943935 |
| B18 | 39 | 2.686181 | 8.276607622 |
| B21 | 44 | 3.059331 | 13.72696646 |
| B23 | 19 | 2.553594 | 6.398119782 |
| B26 | 58 | 2.893861 | 17.0089379 |
| B28 | 77 | 3.825114 | 21.49616883 |
| B29 | 53 | 3.079213 | 22.87507699 |
| B30 | 33 | 1.361654 | 10.58478264 |
| B34 | 42 | 2.781853 | 11.79890764 |
| B38 | 149 | 4.638355 | 41.64297723 |
| B41 | 94 | 3.531574 | 25.69959522 |
| B46 | 47 | 2.374525 | 13.30824383 |
| B48 | 43 | 3.082063 | 11.23051457 |
| B50 | 52 | 2.133259 | 13.83344182 |
| B6 | 34 | 2.539076 | 11.76781346 |
| B7 | 15 | 1.301983 | 6.367585521 |
| B8 | 36 | 3.262412 | 10.20692207 |
| C1 | 120 | 3.968118 | 27.02476312 |
| C11 | 40 | 1.740784 | 10.79315927 |
| C16 | 108 | 4.491135 | 21.17395411 |
| C18 | 97 | 1.284691 | 20.75684331 |
| C21 | 182 | 4.041709 | 37.43562418 |
| C26 | 177 | 3.74193 | 50.70838916 |
| C27 | 95 | 3.112989 | 28.1935755 |
| C31 | 64 | 2.092243 | 19.7041035 |
| C33 | 129 | 2.716845 | 32.50104526 |
| C35 | 127 | 3.918315 | 27.80191245 |
| C36 | 179 | 4.223388 | 44.34466162 |
| C4 | 65 | 2.851841 | 18.84604749 |
| C44 | 154 | 4.014042 | 40.22019178 |
| C46 | 55 | 2.697658 | 15.30836612 |
| C5 | 71 | 1.689708 | 18.6019193 |

| | | | |
|---|---|---|---|
| C6 | 63 | 2.90183 | 16.28147252 |
| C7 | 74 | 1.879246 | 23.48993346 |
| C9 | 53 | 1.64329 | 20.34779867 |
| D1 | 106 | 3.408884 | 25.51556622 |
| D11 | 37 | 1.692155 | 14.72866563 |
| D16 | 130 | 3.463189 | 30.50834223 |
| D18 | 69 | 1.470924 | 22.75495612 |
| D21 | 117 | 3.350747 | 22.14889674 |
| D26 | 74 | 2.308241 | 21.50115611 |
| D27 | 93 | 4.037574 | 18.54898984 |
| D31 | 116 | 2.934773 | 30.87087136 |
| D33 | 73 | 2.62127 | 23.78963001 |
| D35 | 75 | 3.52446 | 13.66063514 |
| D36 | 80 | 2.145638 | 21.07750478 |
| D4 | 67 | 2.503136 | 18.37474395 |
| D44 | 98 | 3.045178 | 23.2410674 |
| D46 | 46 | 3.257327 | 13.16469486 |
| D5 | 83 | 2.52176 | 20.94729684 |
| D6 | 78 | 3.31035 | 28.92706015 |
| D7 | 43 | 2.147724 | 12.36378074 |
| D9 | 62 | 1.451692 | 20.30267782 |

**Table S6 The relative abudances of skin fungi in order level**

| Order | Average relative abundance | A1 | A6 | A7 | A8 | A13 | A17 | A18 | A21 | A23 | A26 | A28 | A29 | A30 | A34 | A38 | A41 | A46 | A48 | A50 | B1 | B13 | B17 | B18 | B21 | B23 | B26 | B28 | B29 | B30 | B34 | B38 | B41 | B46 | B48 | B50 | B6 | B7 | B8 |
|---|---|---|---|---|---|---|---|---|---|---|---|---|---|---|---|---|---|---|---|---|---|---|---|---|---|---|---|---|---|---|---|---|---|---|---|---|---|---|---|
| Malasseziales | 0.774936 | 1 | 0.963 | 0.954 | 0.842 | 0.996 | 0.987 | 0.191 | 0.659 | 0.466 | 0.974 | 0.931 | 0.919 | 0.965 | 0.959 | 0.881 | 0.944 | 0.925 | 0.989 | 0.655 | 0.997 | 0.998 | 0.866 | 0.724 | 0.127 | 0.979 | 0.675 | 0.073 | 0.727 | 0.965 | 0.929 | 0.667 | 0.1 | 0.949 | 0.829 | 0.509 | 0.949 | 0.966 | 0.883 |
| Pleosporales | 0.083797 | 0 | 8E-05 | 0 | 0.114 | 0 | 0 | 0.31 | 0.257 | 0.501 | 6E-04 | 2E-04 | 0.023 | 0.013 | 0.01 | 0.025 | 0.007 | 0.008 | 0.002 | 0.034 | 0.001 | 1E-04 | 0.026 | 0.134 | 0.179 | 0.002 | 0.216 | 0.301 | 0.098 | 0.003 | 0.01 | 0.088 | 0.479 | 3E-04 | 0.116 | 0.007 | 0.017 | 0.021 | 0.02 |
| Capnodiales | 0.06771 | 0 | 0.004 | 3E-04 | 0.01 | 7E-04 | 0 | 0.369 | 0.019 | 0.019 | 0.01 | 0.016 | 0.006 | 0.01 | 0.013 | 0.053 | 0.015 | 0.007 | 0.003 | 0.169 | 0.002 | 0.002 | 0.086 | 0.122 | 0.625 | 0.017 | 0.062 | 0.496 | 0.149 | 0.026 | 0.058 | 0.137 | 0.385 | 0.022 | 0.05 | 0.037 | 0.017 | 0.012 | 0.054 |
| Eurotiales | 0.02805 | 2E-04 | 0.02 | 0.041 | 0.003 | 0.002 | 0.013 | 0.059 | 0.06 | 0.01 | 0.015 | 0.022 | 0.051 | 0.002 | 0.009 | 0.018 | 0.016 | 0.027 | 0.003 | 0.065 | 0 | 0 | 0.017 | 0.018 | 0.057 | 0.002 | 0.018 | 0.018 | 0.004 | 0.004 | 5E-04 | 0.011 | 0.012 | 0.017 | 0.002 | 8E-04 | 0.006 | 0.002 | 0.035 |
| Hypocreales | 0.014065 | 0 | 0.007 | 0 | 0.01 | 0 | 0 | 0.012 | 9E-05 | 0 | 0 | 0.006 | 8E-04 | 0.006 | 0.005 | 0.01 | 0.006 | 0.012 | 0.002 | 0.019 | 0 | 0 | 8E-04 | 0.002 | 0.004 | 0 | 0.006 | 0.027 | 0.001 | 0 | 1E-04 | 0.031 | 0.003 | 0 | 0.002 | 0.003 | 0.006 | 0 | 0.002 |
| Fungi unidentified | 0.007599 | 0 | 0 | 0 | 0.01 | 0 | 0 | 0.007 | 0 | 3E-04 | 0 | 0.001 | 3E-04 | 0 | 1E-04 | 1E-03 | 7E-04 | 0.005 | 3E-04 | 0.002 | 0 | 0 | 0 | 0 | 0.002 | 0 | 0 | 0.005 | 0.004 | 0 | 0 | 0.004 | 0.004 | 0.005 | 2E-04 | 0.441 | 0 | 0 | 0 |
| Saccharomycetales | 0.002918 | 0 | 0 | 0 | 0 | 0 | 0 | 0 | 0 | 5E-05 | 0 | 4E-04 | 0 | 0.004 | 0 | 0 | 0 | 0 | 0 | 0.001 | 0 | 0 | 6E-04 | 0 | 0 | 0 | 0 | 0 | 0 | 0 | 0 | 8E-04 | 0 | 0 | 0 | 0 | 0 | 0 | 1E-04 |
| Filobasidiales | 0.002381 | 0 | 2E-04 | 0 | 0 | 0 | 0 | 0.005 | 0.001 | 0.001 | 0 | 3E-04 | 0 | 1E-04 | 2E-04 | 0 | 0 | 0.005 | 2E-04 | 0.017 | 0 | 0 | 0.002 | 0 | 9E-04 | 0 | 0.003 | 2E-04 | 3E-04 | 0 | 0 | 0 | 0.001 | 0 | 2E-04 | 0 | 0 | 0 | 5E-04 |
| Dothideales | 0.002198 | 0 | 0 | 0 | 3E-04 | 0 | 0 | 1E-03 | 0 | 0 | 0 | 2E-04 | 0 | 0 | 0 | 0 | 0.001 | 0 | 0 | 0 | 0 | 0 | 6E-04 | 0 | 1E-04 | 0 | 3E-04 | 0.021 | 0 | 0 | 1E-04 | 4E-04 | 0.001 | 0 | 0 | 1E-04 | 2E-04 | 0 | 0 |
| Pezizales | 0.00196 | 0 | 0 | 6E-04 | 0 | 4E-04 | 0 | 0.002 | 0 | 2E-04 | 0 | 0 | 0 | 0 | 0 | 0 | 0 | 0 | 0 | 0 | 0 | 0 | 0 | 0 | 7E-04 | 0 | 0 | 0 | 0 | 0 | 0 | 0.001 | 0 | 9E-04 | 0 | 0 | 4E-04 | 0 | 0 |
| Trichosphaeriales | 0.001441 | 0 | 0 | 0.001 | 0 | 0 | 0 | 0.016 | 0 | 5E-04 | 0 | 0.018 | 0 | 0 | 0 | 0 | 0 | 0 | 0 | 0 | 0 | 0 | 1E-04 | 0 | 0 | 0 | 0 | 0 | 0 | 2E-04 | 0 | 0 | 0 | 1E-04 | 0 | 0 | 0 | 0 | 0 |
| Agaricales | 0.001351 | 0 | 0 | 0 | 0 | 0 | 0 | 0 | 0 | 0 | 0 | 7E-04 | 0 | 0 | 0 | 0 | 0.003 | 0.001 | 0 | 0 | 0 | 0 | 0 | 0 | 0 | 0 | 0.007 | 0.001 | 1E-03 | 0 | 4E-04 | 0.018 | 0.002 | 0.002 | 4E-04 | 0.001 | 0 | 0 | 0 |
| Tremellales | 0.00098 | 0 | 2E-04 | 1E-04 | 0 | 0 | 0 | 0.004 | 0 | 9E-04 | 0 | 2E-04 | 0 | 0 | 0 | 0 | 0 | 0 | 2E-04 | 0 | 0 | 0 | 0 | 0 | 0 | 0 | 0.003 | 0 | 0 | 0 | 0 | 0.002 | 6E-05 | 0 | 0 | 0 | 0 | 0 | 0 |
| Sordariales | 0.000861 | 0 | 0 | 0 | 0 | 0 | 0 | 0 | 0 | 0 | 0 | 0 | 0 | 0 | 0 | 0 | 0 | 0 | 0 | 0.002 | 0 | 1E-04 | 1E-04 | 0 | 0 | 0 | 0.003 | 0.003 | 0 | 7E-04 | 0 | 0.003 | 2E-04 | 0 | 0 | 0 | 0 | 0 | 0 |
| Glomerellales | 0.000847 | 0 | 0 | 0 | 5E-04 | 0 | 0 | 0 | 0 | 0 | 0 | 1E-04 | 0 | 0 | 1E-04 | 0.006 | 0 | 0 | 0 | 0 | 0 | 0 | 0 | 0 | 4E-04 | 0 | 0 | 0.027 | 0.012 | 0 | 0 | 0.002 | 0.002 | 0 | 0 | 0 | 0 | 0 | 0 |
| Sporidiobolales | 0.000797 | 0 | 0.001 | 0 | 0 | 0 | 0 | 0 | 0 | 0 | 0 | 1E-04 | 0 | 0 | 0 | 0 | 0.001 | 3E-04 | 0 | 0 | 0 | 0 | 0 | 0 | 0 | 0 | 0.005 | 2E-04 | 0 | 0 | 0 | 0 | 0.004 | 8E-04 | 4E-04 | 0 | 0 | 0 | 0 |
| Polyporales | 0.000747 | 0 | 0 | 0 | 0 | 3E-04 | 0 | 0 | 0 | 0 | 0 | 6E-04 | 0 | 1E-04 | 3E-04 | 0 | 0 | 0 | 3E-04 | 0.006 | 0 | 0 | 0 | 0 | 0 | 0 | 2E-04 | 0.003 | 0.001 | 0 | 3E-04 | 0.01 | 8E-04 | 1E-03 | 0 | 1E-03 | 0 | 0 | 0 |
| Chaetothyriales | 0.000667 | 0 | 5E-05 | 0 | 0.007 | 0 | 0 | 5E-04 | 5E-04 | 0 | 0 | 7E-04 | 0 | 0 | 2E-04 | 0 | 0 | 8E-04 | 0 | 0.007 | 0 | 0 | 8E-04 | 0 | 4E-04 | 0 | 0.001 | 0.002 | 0 | 0 | 2E-04 | 0.001 | 0.002 | 6E-04 | 0 | 0 | 0.004 | 0 | 0 |
| Wallemiales | 0.00061 | 0 | 0 | 0 | 0 | 0 | 0 | 3E-04 | 0 | 0 | 0 | 3E-04 | 0 | 0 | 0 | 0 | 0 | 0 | 0 | 0 | 0 | 0 | 0 | 0 | 0 | 0 | 0 | 0 | 0 | 0 | 0 | 0 | 0 | 0 | 0 | 0 | 0 | 0 | 0 |
| Xylariales | 0.000581 | 0 | 0 | 0 | 0.001 | 0 | 0 | 0 | 0 | 0 | 0 | 0 | 0 | 0 | 0 | 0.005 | 0 | 0 | 0 | 0 | 0 | 0 | 1E-04 | 0 | 1E-04 | 0 | 0.001 | 3E-04 | 0 | 0 | 1E-04 | 0 | 3E-04 | 0.001 | 0 | 0 | 0 | 0 | 0 |
| Erysiphales | 0.000484 | 0 | 0 | 0 | 0 | 0 | 0 | 0.001 | 0 | 0 | 0 | 0 | 0 | 0 | 8E-04 | 0 | 0 | 0 | 0 | 0 | 0 | 0 | 0 | 0 | 0 | 0 | 0 | 0.017 | 0 | 0 | 4E-04 | 0 | 0 | 0 | 0 | 7E-04 | 0 | 0 | 0 |
| Entomophthorales | 0.000473 | 0 | 0 | 0 | 0 | 0 | 0 | 0 | 0 | 0 | 0 | 0 | 0 | 0 | 0 | 0 | 0 | 0 | 0 | 0 | 0 | 0 | 0 | 0 | 0 | 0 | 0 | 0 | 0 | 0 | 0 | 0 | 0 | 0 | 0 | 0 | 0 | 0 | 0 |
| Botryosphaeriales | 0.000462 | 0 | 0.002 | 0 | 0 | 0 | 0 | 0 | 0 | 0 | 0 | 0 | 0 | 0 | 0 | 0 | 0 | 0 | 0 | 0.018 | 0 | 0 | 0 | 0 | 0 | 0 | 0 | 0 | 0 | 0 | 0 | 0 | 0 | 0 | 0 | 0 | 0 | 0 | 0 |
| Cystofilobasidiales | 0.000455 | 0 | 0 | 0 | 0 | 0 | 0 | 0.003 | 0 | 0 | 0 | 0 | 6E-04 | 0 | 0 | 2E-04 | 0 | 8E-04 | 7E-04 | 0 | 0 | 0 | 0 | 0 | 0 | 0 | 0 | 0 | 0 | 0 | 0 | 0 | 0 | 0 | 0 | 0 | 0 | 0 | 0 |
| Ascomycota unidentified | 0.000359 | 0 | 0 | 0 | 8E-04 | 0 | 0 | 0.003 | 2E-04 | 0 | 0 | 0 | 0 | 0 | 0 | 0 | 0.002 | 0.006 | 0 | 0 | 0 | 0 | 0 | 0 | 0.001 | 0 | 0 | 0.003 | 0 | 0 | 0 | 7E-04 | 0 | 5E-04 | 0 | 0 | 0 | 0 | 0 |
| Dothideomycetes unidentified | 0.00028 | 0 | 0.002 | 0 | 0 | 0 | 0 | 0.01 | 0.001 | 0 | 0 | 0 | 0 | 0 | 0 | 0 | 0 | 0 | 0 | 0 | 0 | 0 | 2E-04 | 0 | 0 | 0 | 0 | 0 | 0 | 0 | 0 | 0.001 | 0 | 0 | 0 | 0 | 0 | 0 | 0.003 |
| Erythrobasidiales | 0.000239 | 0 | 0 | 0 | 0 | 0 | 0 | 0.002 | 0 | 0 | 0 | 0 | 0 | 0 | 0 | 0 | 0 | 0 | 0 | 0 | 0 | 0 | 0 | 0 | 0.001 | 0 | 0 | 0 | 0 | 0 | 0 | 0 | 5E-04 | 0 | 0 | 0 | 0 | 0 | 0 |
| Trichosporonales | 0.000195 | 0 | 0 | 0 | 0 | 0 | 0 | 9E-04 | 0 | 0 | 8E-05 | 0 | 0 | 0 | 0 | 0 | 0 | 0 | 0 | 0 | 0 | 0 | 0 | 0 | 0 | 0 | 0 | 0 | 0 | 0 | 0 | 0 | 0 | 0 | 0 | 0 | 0 | 0 | 0 |
| Cystobasidiales | 0.000177 | 0 | 0 | 0 | 0 | 0 | 0 | 2E-04 | 0 | 0 | 0 | 0 | 0 | 0 | 0 | 0 | 0 | 0 | 0 | 0 | 0 | 0 | 0 | 0 | 0 | 0 | 0 | 0 | 0 | 0 | 0 | 0 | 4E-04 | 0 | 0 | 0 | 0 | 0 | 0 |
| Helotiales | 0.000176 | 0 | 0 | 0 | 5E-04 | 0 | 0 | 0 | 6E-04 | 0 | 0 | 2E-04 | 0 | 0 | 0 | 0 | 0.003 | 0 | 0 | 0 | 0 | 0 | 0 | 0 | 0 | 0 | 0 | 0 | 0 | 0 | 0 | 0.003 | 0 | 0 | 0 | 0 | 0 | 0 | 0 |
| Tubeufiales | 0.000169 | 0 | 0 | 0 | 0 | 0 | 0 | 0 | 0.002 | 0 | 0 | 0 | 0 | 0 | 0 | 0 | 0 | 0 | 0 | 0 | 0 | 0 | 2E-04 | 0 | 3E-04 | 0 | 0 | 0 | 0 | 0 | 1E-03 | 0 | 0 | 0 | 0 | 0 | 0 | 0 | 0.003 |
| Cantharellales | 0.000163 | 0 | 0 | 0 | 0 | 0 | 0 | 0 | 0 | 0 | 0 | 0 | 2E-04 | 0 | 2E-04 | 0 | 0 | 1E-03 | 0 | 0 | 0 | 0 | 0 | 0 | 0 | 0 | 0 | 0 | 0.002 | 0 | 0 | 0.001 | 0 | 0 | 0 | 0 | 0 | 0 | 0 |
| Ustilaginales | 0.000129 | 0 | 0 | 0 | 0 | 0 | 0 | 0 | 0 | 0 | 0 | 0 | 0 | 0 | 0 | 0 | 0 | 0 | 0 | 0 | 0 | 0 | 0 | 0 | 0 | 0 | 0 | 0 | 2E-04 | 0 | 0 | 0.002 | 7E-04 | 0 | 0 | 2E-04 | 0 | 0 | 0 |
| Mortierellales | 0.000126 | 0 | 0 | 0 | 0 | 0 | 0 | 0 | 0 | 0 | 0 | 0 | 0 | 0 | 0 | 0 | 0 | 0 | 0 | 5E-04 | 0 | 0 | 0 | 0 | 0 | 0 | 0 | 0 | 0 | 0 | 0 | 0 | 0 | 0 | 0 | 0 | 6E-04 | 0 | 0 |
| Thelebolales | 0.000126 | 0 | 0 | 0 | 0 | 0 | 0 | 4E-04 | 0 | 0 | 0 | 0 | 0 | 0 | 0 | 0 | 0 | 0 | 0 | 0.004 | 0 | 0 | 0 | 0 | 0 | 0 | 0 | 0 | 0 | 0 | 0 | 0.002 | 0 | 0 | 0 | 0 | 0 | 0 | 0 |
| Agaricostilbales | 0.000121 | 0 | 0 | 0 | 0 | 0 | 0 | 0 | 0 | 0 | 0 | 0 | 0 | 0 | 0 | 0 | 0 | 0 | 0 | 0 | 0 | 0 | 0 | 0 | 0 | 0 | 0 | 0 | 0 | 0 | 0 | 0.002 | 0 | 0 | 0 | 0 | 0 | 0 | 0 |
| Cystobasidiomycetes unidentified | 0.000121 | 0 | 0 | 0 | 0 | 0 | 0 | 0 | 0 | 0 | 0 | 0 | 0 | 0 | 0 | 0 | 0 | 0 | 0 | 0 | 0 | 0 | 0 | 0 | 0 | 0 | 0 | 0 | 0 | 0 | 0 | 0 | 0 | 0 | 0 | 0 | 0 | 0 | 0 |
| Basidiomycota unidentified | 0.000117 | 0 | 0 | 0 | 0 | 0 | 0 | 0 | 0 | 0 | 0 | 0 | 0 | 0 | 0 | 0 | 0 | 0 | 0 | 0 | 0 | 0 | 0 | 0 | 0 | 0 | 0 | 0 | 0 | 0 | 0 | 0.002 | 0 | 0 | 0 | 0 | 0 | 0 | 0 |
| Microbotryomycetes unidentified | 0.00016 | 0 | 0 | 0 | 0 | 0 | 0 | 0 | 0 | 0 | 0 | 0 | 0 | 0 | 0 | 0 | 0 | 0 | 0 | 0 | 0 | 0 | 0 | 0 | 0 | 0 | 0 | 0 | 0 | 0 | 0 | 0 | 0 | 0 | 0 | 0 | 0 | 0 | 0 |
| Unassigned | 9.63E-05 | 0 | 0 | 0 | 5E-04 | 0 | 0 | 0 | 0 | 0 | 0 | 4E-04 | 0 | 0 | 0 | 0 | 0 | 0 | 0 | 0 | 0 | 0 | 0 | 0 | 0 | 0 | 0 | 0 | 0 | 0 | 0 | 0 | 0 | 0 | 0 | 0 | 0 | 0 | 0 |
| Russulales | 6.63E-05 | 0 | 0 | 0 | 0 | 0 | 0 | 0 | 0 | 0 | 0 | 0 | 0 | 0 | 0 | 0 | 0 | 0 | 0 | 0 | 0 | 0 | 0 | 0 | 0 | 0 | 0 | 0 | 0 | 4E-04 | 2E-04 | 0 | 0 | 0 | 0 | 0 | 0 | 0 | 0 |
| Agaricomycetes unidentified | 6.12E-05 | 0 | 0 | 0 | 0 | 0 | 0 | 0 | 0 | 0 | 0 | 0 | 0 | 0 | 0 | 0 | 0 | 0 | 0 | 0 | 0 | 0 | 0 | 0 | 0 | 0 | 0 | 0 | 0 | 0 | 0 | 0 | 0 | 0 | 0 | 0 | 0 | 0 | 0 |
| Rozellomycota unidentified | 5.9E-05 | 0 | 0 | 0 | 0 | 0 | 0 | 0 | 0 | 0 | 0 | 0 | 0 | 0 | 0 | 0 | 0 | 0 | 0 | 0 | 0 | 0 | 0 | 0 | 0 | 0 | 0 | 0 | 0 | 0 | 0 | 0.002 | 0 | 0 | 0 | 0 | 0 | 0 | 0 |
| Diaporthales | 5.01E-05 | 0 | 0 | 0 | 0 | 0 | 0 | 0 | 0 | 0 | 0 | 0 | 0 | 0 | 0 | 0 | 0 | 0 | 0 | 0 | 0 | 0 | 0 | 0 | 0 | 0 | 0 | 0 | 0 | 0 | 0 | 0 | 0 | 0 | 0 | 0 | 0 | 0 | 0 |
| Sordariomycetes unidentified | 4.87E-05 | 0 | 0 | 0 | 4E-04 | 5E-05 | 0 | 0 | 0 | 0 | 0 | 0 | 0 | 0 | 0 | 0 | 0 | 0 | 0 | 0 | 0 | 0 | 0 | 0 | 0 | 0 | 0 | 0 | 0 | 0 | 0 | 4E-04 | 0 | 0 | 0 | 0 | 0 | 0 | 0 |
| Lichenostigmatales | 4.84E-05 | 0 | 0 | 0 | 2E-04 | 0 | 0 | 0 | 0 | 0 | 0 | 0 | 0 | 0 | 0 | 0 | 0 | 0 | 0 | 0 | 0 | 0 | 0 | 0 | 0 | 0 | 0 | 0 | 0 | 0 | 0 | 0 | 0 | 0 | 0 | 0 | 0 | 0 | 0 |
| Tremellomycetes unidentified | 4.19E-05 | 0 | 0 | 0 | 0 | 0 | 0 | 0 | 0 | 0 | 0 | 0 | 0 | 0 | 0 | 0 | 0 | 0 | 0 | 0 | 0 | 0 | 0 | 0 | 0 | 0 | 0 | 0 | 0 | 0 | 0 | 0 | 0 | 0 | 0 | 0 | 0 | 0 | 0 |
| Leucosporidiales | 4.04E-05 | 0 | 0 | 0.003 | 0 | 0 | 0 | 0 | 0 | 0 | 0 | 0 | 0 | 0 | 0 | 0 | 0 | 0 | 0 | 0 | 0 | 0 | 0 | 0 | 0 | 0 | 0 | 0 | 0 | 0 | 0 | 3E-04 | 0 | 0 | 0 | 0 | 0 | 0 | 0 |
| Auriculariales | 3.71E-05 | 0 | 0 | 0 | 0 | 0 | 0 | 0 | 0 | 0 | 0 | 0 | 0 | 0 | 0 | 0 | 0 | 0 | 0 | 0 | 0 | 0 | 0 | 0 | 0 | 0 | 0 | 0.002 | 0 | 0 | 0 | 0 | 0 | 0 | 0 | 0 | 0 | 0 | 0 |
| Cryptomycota unidentified | 3.62E-05 | 0 | 0 | 0 | 0 | 0 | 0 | 0 | 0 | 0 | 0 | 0 | 0 | 0 | 0 | 0 | 0 | 0 | 0 | 0 | 0 | 0 | 0 | 0 | 0 | 0 | 0 | 0 | 0 | 0 | 0 | 0.002 | 0 | 0 | 0 | 0 | 0 | 0 | 0 |
| Microascales | 3.51E-05 | 0 | 0 | 0 | 0 | 0 | 0 | 0 | 0 | 0 | 0 | 0 | 0 | 0 | 2E-04 | 0 | 0 | 0 | 0 | 0 | 0 | 0 | 0 | 0 | 0 | 0 | 0 | 0 | 0 | 0 | 0 | 0 | 0 | 0 | 0 | 0 | 0 | 0 | 0 |
| Pezizomycetes unidentified | 3.42E-05 | 0 | 0 | 0 | 0 | 0 | 0 | 0.003 | 0 | 0 | 0 | 0 | 0 | 0 | 0 | 0 | 0 | 0 | 0 | 0 | 0 | 0 | 0 | 0 | 0 | 0 | 0 | 0 | 0 | 0 | 0 | 0 | 0 | 0 | 0 | 0 | 0 | 0 | 0 |
| Onygenales | 3.12E-05 | 0 | 0 | 0 | 0 | 0 | 0 | 0 | 0 | 0 | 0 | 0.001 | 0 | 0 | 9E-04 | 0 | 0 | 0 | 0 | 0 | 0 | 0 | 0 | 0 | 0 | 0 | 0 | 0 | 0 | 0 | 0 | 0 | 0 | 0 | 0 | 0 | 0 | 0 | 0 |
| Mucoromycota unidentified | 2.45E-05 | 0 | 0 | 0 | 0 | 0 | 0 | 0 | 0 | 0 | 0 | 0 | 0 | 0 | 0 | 0 | 0 | 0 | 0 | 0 | 0 | 0 | 0 | 0 | 0 | 0 | 0 | 0 | 0 | 0 | 0 | 0.002 | 0 | 0 | 0 | 0 | 0 | 0 | 0 |
| Golubeviales | 2.35E-05 | 0 | 0 | 0 | 0 | 0 | 0 | 0 | 0 | 0 | 0 | 0 | 0 | 0 | 0 | 0 | 0 | 0 | 0 | 0 | 0 | 0 | 0 | 0 | 0 | 0 | 0 | 0 | 0 | 0 | 0 | 0 | 0 | 0 | 0 | 0 | 0 | 0 | 0 |
| Corticiales | 2.33E-05 | 0 | 0 | 0 | 0 | 0 | 0 | 0 | 0 | 0 | 0 | 0 | 0 | 0 | 0 | 0 | 0 | 0 | 0 | 0 | 0 | 0 | 0 | 0 | 0 | 0 | 0 | 0 | 0 | 0 | 0 | 0 | 0 | 0 | 0 | 0 | 0 | 0 | 0 |
| Mycosphaerellales | 2.31E-05 | 0 | 0 | 0 | 0 | 0 | 0 | 0 | 0 | 0 | 0 | 0 | 0 | 0 | 0 | 0 | 0 | 0 | 0 | 0 | 0 | 0 | 0 | 0 | 0 | 0 | 0 | 0 | 0 | 0 | 0 | 4E-04 | 0.001 | 0 | 0 | 0 | 0 | 0 | 0 |
| Pezizomycotina unidentified | 2.07E-05 | 0 | 0 | 0 | 0 | 0 | 0 | 0 | 0 | 0 | 0 | 0 | 0 | 0 | 0 | 0 | 0 | 0 | 0 | 0 | 0 | 0 | 0 | 0 | 0 | 0 | 0 | 0 | 0 | 0 | 0 | 0 | 0 | 0 | 0 | 0 | 0 | 0 | 0 |
| Myrmecridiales | 1.96E-05 | 0 | 0 | 0 | 0 | 0 | 0 | 0 | 0 | 0 | 0 | 0 | 0 | 0 | 0 | 0 | 0 | 0 | 0 | 0 | 0 | 0 | 0 | 0 | 0 | 0 | 0 | 0 | 0 | 0 | 0 | 0 | 0 | 0 | 0 | 0 | 0 | 0 | 0 |
| Venturiales | 1.78E-05 | 0 | 0 | 0 | 0 | 0 | 0 | 0 | 0 | 0 | 0 | 0 | 0 | 0 | 0 | 0 | 0 | 0 | 0 | 4E-04 | 0 | 0 | 0 | 0 | 0 | 0 | 0 | 0 | 0 | 0 | 0 | 0 | 0 | 0 | 0 | 0 | 0 | 0 | 0 |
| Myriangiales | 1.76E-05 | 0 | 0 | 0 | 0 | 0 | 0 | 0 | 0 | 0 | 0 | 0 | 0 | 0 | 0 | 0 | 0 | 0 | 0 | 0 | 0 | 0 | 0 | 0 | 0 | 0 | 0 | 0 | 0 | 0.001 | 0 | 0 | 0 | 0 | 0 | 0 | 0 | 0 | 0 |
| Exobasidiales | 1.43E-05 | 0 | 0 | 0 | 0 | 0 | 0 | 0 | 0 | 0 | 0 | 0 | 0 | 0 | 0 | 0 | 0 | 0 | 0 | 0 | 0 | 0 | 0 | 0 | 0 | 0 | 0 | 0 | 0 | 0 | 0 | 0 | 0 | 0 | 0 | 0 | 0 | 0 | 0 |
| Pyxidiophorales | 1.19E-05 | 0 | 0 | 0 | 0 | 0 | 0 | 0 | 0 | 0 | 0 | 0 | 0 | 0 | 0 | 0 | 0 | 0 | 0 | 0 | 0 | 0 | 0 | 0 | 0 | 0 | 0 | 0 | 0 | 0 | 0 | 0 | 0 | 0 | 0 | 0 | 0 | 0 | 0 |
| Orbiliales | 8.31E-06 | 0 | 0 | 0 | 0 | 0 | 0 | 0 | 0 | 0 | 0 | 0 | 0 | 0 | 0 | 0 | 0 | 0 | 0 | 0 | 0 | 0 | 0 | 0 | 0 | 0 | 0 | 0 | 0 | 0 | 0 | 6E-04 | 0 | 0 | 0 | 0 | 0 | 0 | 0 |
| Lulworthiales | 7.48E-06 | 0 | 0 | 0 | 0 | 0 | 0 | 0 | 0 | 0 | 0 | 0 | 0 | 0 | 0 | 0 | 0 | 0 | 0 | 0 | 0 | 0 | 0 | 0 | 0 | 0 | 0 | 0 | 0 | 0 | 0 | 6E-04 | 0 | 0 | 0 | 0 | 0 | 0 | 0 |
| Mucorales | 7.19E-06 | 0 | 0 | 0 | 0 | 0 | 0 | 0 | 0 | 0 | 0 | 0 | 0 | 0 | 0 | 0 | 0 | 0 | 0 | 0 | 0 | 0 | 0 | 0 | 0 | 0 | 0 | 0 | 0 | 0 | 0 | 0 | 0 | 0 | 0 | 0 | 0 | 0 | 0 |
| Taphrinales | 6.3E-06 | 0 | 0 | 0 | 0 | 0 | 0 | 0 | 0 | 0 | 0 | 0 | 0 | 0 | 0 | 0 | 0 | 0 | 0 | 0 | 0 | 0 | 0 | 0 | 0 | 0 | 0 | 0 | 0 | 0 | 0 | 0 | 0 | 0 | 0 | 0 | 0 | 0 | 0 |

| | | | | | | | | | | | | | | | | | | | | | | | | | | | | | | | | | | | | | | | |
|---|---|---|---|---|---|---|---|---|---|---|---|---|---|---|---|---|---|---|---|---|---|---|---|---|---|---|---|---|---|---|---|---|---|---|---|---|---|---|---|
| Ascomycota GS35 | 6.21E-06 | 0 | 0 | 0 | 0 | 0 | 0 | 0 | 0 | 0 | 0 | 0 | 0 | 0 | 0 | 0 | 0 | 0 | 0 | 0 | 0 | 0 | 0 | 0 | 0 | 0 | 0 | 0 | 0 | 0 | 0 | 0 | 0 | 0 | 0 | 0 | 0 | 0 | 0 |
| Atheliales | 5.82E-06 | 0 | 0 | 0 | 0 | 0 | 0 | 0 | 0 | 0 | 0 | 0 | 0 | 0 | 0 | 0 | 0 | 0 | 0 | 0 | 0 | 0 | 0 | 0 | 0 | 0 | 0 | 0 | 0 | 0 | 0 | 4E-04 | 0 | 0 | 0 | 0 | 0 | 0 | 0 |
| Thelephorales | 5.73E-06 | 0 | 0 | 0 | 0 | 0 | 0 | 0 | 0 | 0 | 0 | 0 | 0 | 0 | 0 | 0 | 0 | 0 | 0 | 0 | 0 | 0 | 0 | 0 | 0 | 0 | 0 | 0 | 0 | 0 | 0 | 0 | 0 | 0 | 0 | 0 | 0 | 0 | 0 |
| Archaeorhizomycetales | 5.5E-06 | 0 | 0 | 0 | 0 | 0 | 0 | 0 | 0 | 0 | 0 | 0 | 0 | 0 | 0 | 0 | 0 | 0 | 0 | 0 | 0 | 0 | 0 | 0 | 0 | 0 | 0 | 0 | 0 | 0 | 0 | 0 | 0 | 0 | 0 | 0 | 0 | 0 | 0 |
| Gloeophyllales | 5.11E-06 | 0 | 0 | 0 | 0 | 0 | 0 | 0 | 0 | 0 | 0 | 0 | 0 | 0 | 0 | 0 | 0 | 0 | 0 | 0 | 0 | 0 | 0 | 0 | 0 | 0 | 0 | 0 | 0 | 0 | 0 | 0 | 0 | 0 | 0 | 0 | 0 | 0 | 0 |
| Leotiomycetes unidentified | 4.57E-06 | 0 | 0 | 0 | 0 | 0 | 0 | 0 | 0 | 0 | 0 | 0 | 0 | 0 | 0 | 0 | 0 | 0 | 0 | 0 | 0 | 0 | 0 | 0 | 0 | 0 | 0 | 0 | 0 | 0 | 0 | 4E-04 | 0 | 0 | 0 | 0 | 0 | 0 | 0 |
| Hymenochaetales | 4.01E-06 | 0 | 0 | 0 | 0 | 0 | 0 | 0 | 0 | 0 | 0 | 0 | 0 | 0 | 0 | 0 | 0 | 0 | 0 | 0 | 0 | 0 | 0 | 0 | 0 | 0 | 0 | 0 | 0 | 0 | 0 | 0 | 0 | 0 | 0 | 0 | 0 | 0 | 0 |
| Eurotiomycetes unidentified | 3.87E-06 | 0 | 0 | 0 | 0 | 0 | 0 | 0 | 0 | 0 | 0 | 0 | 0 | 0 | 0 | 0 | 0 | 0 | 0 | 0 | 0 | 0 | 0 | 0 | 0 | 0 | 0 | 0 | 0 | 0 | 0 | 0 | 0 | 0 | 0 | 0 | 0 | 0 | 0 |
| Rhizophlyctidales | 3.54E-06 | 0 | 0 | 0 | 0 | 0 | 0 | 0 | 0 | 0 | 0 | 0 | 0 | 0 | 0 | 0 | 0 | 0 | 0 | 0 | 0 | 0 | 0 | 0 | 0 | 0 | 0 | 0 | 0 | 0 | 0 | 0 | 0 | 0 | 0 | 0 | 0 | 0 | 0 |
| Melanosporales | 2.87E-06 | 0 | 0 | 0 | 0 | 0 | 0 | 0 | 0 | 0 | 0 | 0 | 0 | 0 | 0 | 0 | 0 | 0 | 0 | 0 | 0 | 0 | 0 | 0 | 0 | 0 | 0 | 0 | 0 | 0 | 0 | 0 | 0 | 0 | 0 | 0 | 0 | 0 | 0 |
| Kriegeriales | 2.86E-06 | 0 | 0 | 0 | 0 | 0 | 0 | 0 | 0 | 0 | 0 | 0 | 0 | 0 | 0 | 0 | 0 | 0 | 0 | 0 | 0 | 0 | 0 | 0 | 0 | 0 | 0 | 0 | 0 | 0 | 0 | 0 | 0 | 0 | 0 | 0 | 0 | 0 | 0 |
| Chytridiomycota unidentified | 2.86E-06 | 0 | 0 | 0 | 0 | 0 | 0 | 0 | 0 | 0 | 0 | 0 | 0 | 0 | 0 | 0 | 0 | 0 | 0 | 0 | 0 | 0 | 0 | 0 | 0 | 0 | 0 | 0 | 0 | 0 | 0 | 0 | 0 | 0 | 0 | 0 | 0 | 0 | 0 |
| Rozellomycotina GS11 | 2.36E-06 | 0 | 0 | 0 | 0 | 0 | 0 | 0 | 0 | 0 | 0 | 0 | 0 | 0 | 0 | 0 | 0 | 0 | 0 | 0 | 0 | 0 | 0 | 0 | 0 | 0 | 0 | 0 | 0 | 0 | 0 | 0 | 0 | 0 | 0 | 0 | 0 | 0 | 0 |
| Lecanorales | 1.65E-06 | 0 | 0 | 0 | 0 | 0 | 0 | 0 | 0 | 1E-04 | 0 | 0 | 0 | 0 | 0 | 0 | 0 | 0 | 0 | 0 | 0 | 0 | 0 | 0 | 0 | 0 | 0 | 0 | 0 | 0 | 0 | 0 | 0 | 0 | 0 | 0 | 0 | 0 | 0 |
| Glomeromycetes unidentified | 1.15E-06 | 0 | 0 | 0 | 0 | 0 | 0 | 0 | 0 | 0 | 0 | 0 | 0 | 0 | 0 | 0 | 0 | 0 | 0 | 0 | 0 | 0 | 0 | 0 | 0 | 0 | 0 | 0 | 0 | 0 | 0 | 0 | 0 | 0 | 0 | 0 | 0 | 0 | 0 |
| Entylomatales | 1.09E-06 | 0 | 0 | 0 | 0 | 0 | 0 | 0 | 0 | 0 | 0 | 0 | 0 | 0 | 0 | 0 | 0 | 0 | 0 | 0 | 0 | 0 | 0 | 0 | 0 | 0 | 0 | 0 | 0 | 0 | 0 | 0 | 0 | 0 | 0 | 0 | 0 | 0 | 0 |
| Tilletiales | 8.13E-07 | 0 | 0 | 0 | 0 | 0 | 0 | 0 | 0 | 0 | 0 | 0 | 0 | 0 | 0 | 0 | 0 | 0 | 0 | 0 | 0 | 0 | 0 | 0 | 0 | 0 | 0 | 0 | 0 | 0 | 0 | 0 | 0 | 0 | 0 | 0 | 0 | 0 | 0 |
| Trechisporales | 4.29E-07 | 0 | 0 | 0 | 0 | 0 | 0 | 0 | 0 | 0 | 0 | 0 | 0 | 0 | 0 | 0 | 0 | 0 | 0 | 0 | 0 | 0 | 0 | 0 | 0 | 0 | 0 | 0 | 0 | 0 | 0 | 0 | 0 | 0 | 0 | 0 | 0 | 0 | 0 |

| Order | Average relative abundance | C1 | C4 | C5 | C6 | C7 | C9 | C11 | C16 | C18 | C21 | C26 | C27 | C31 | C33 | C35 | C36 | C44 | C46 | D1 | D4 | D5 | D6 | D7 | D9 | D11 | D16 | D18 | D21 | D26 | D27 | D31 | D33 | D35 | D36 | D44 | D46 |
|---|---|---|---|---|---|---|---|---|---|---|---|---|---|---|---|---|---|---|---|---|---|---|---|---|---|---|---|---|---|---|---|---|---|---|---|---|---|
| Malasseziales | 0.774936 | 0.428 | 0.663 | 0.864 | 0.874 | 0.857 | 0.877 | 0.945 | 0.377 | 0.923 | 0.315 | 0.601 | 0.589 | 0.911 | 0.831 | 0.713 | 0.29 | 0.568 | 0.771 | 0.719 | 0.819 | 0.873 | 0.745 | 0.935 | 0.95 | 0.958 | 0.708 | 0.924 | 0.676 | 0.758 | 0.398 | 0.9 | 0.875 | 0.791 | 0.827 | 0.78 | 0.868 |
| Pleosporales | 0.083797 | 0.246 | 0.038 | 0.013 | 0.025 | 0.03 | 0.057 | 0.023 | 0.179 | 0.024 | 0.235 | 0.145 | 0.093 | 0.06 | 0.079 | 0.064 | 0.168 | 0.189 | 0.179 | 0.091 | 0.08 | 0.044 | 0.153 | 0.026 | 0.008 | 0.034 | 0.113 | 0.03 | 0.107 | 0.202 | 0.279 | 0.026 | 0.064 | 0.047 | 0.059 | 0.046 | 0.041 |
| Capnodiales | 0.06771 | 0.149 | 0.125 | 0.045 | 0.018 | 0.028 | 0.013 | 0.007 | 0.185 | 0.022 | 0.137 | 0.088 | 0.058 | 0.011 | 0.039 | 0.077 | 0.142 | 0.104 | 0.011 | 0.041 | 0.014 | 0.037 | 0.035 | 0.012 | 0.012 | 0.003 | 0.098 | 0.023 | 0.119 | 0.018 | 0.082 | 0.013 | 0.022 | 0.094 | 0.051 | 0.066 | 0.019 |
| Eurotiales | 0.02805 | 0.046 | 0.033 | 0.008 | 0.005 | 0.01 | 0.002 | 0.005 | 0.03 | 0.012 | 0.155 | 0.103 | 0.216 | 0.008 | 0.022 | 0.072 | 0.255 | 0.041 | 0.01 | 0.071 | 0.03 | 0.013 | 0.022 | 0.009 | 0.005 | 0.003 | 0.038 | 0.004 | 0.03 | 0.005 | 0.088 | 0.006 | 0.013 | 0.028 | 0.029 | 0.028 | 0.014 |
| Hypocreales | 0.014065 | 0.064 | 0.05 | 0.005 | 0.005 | 0.012 | 0.014 | 0.007 | 0.131 | 0.007 | 0.053 | 0.015 | 0.01 | 0.004 | 0.015 | 0.038 | 0.056 | 0.045 | 0.017 | 0.039 | 0.024 | 0.011 | 0.012 | 0.004 | 0.017 | 0.001 | 0.012 | 0.008 | 0.027 | 0.006 | 0.07 | 0.022 | 0.011 | 0.01 | 0.011 | 0.01 | 0.022 |
| Fungi unidentified | 0.007599 | 1E-03 | 2E-04 | 0.031 | 0.004 | 8E-04 | 9E-04 | 3E-04 | 3E-04 | 1E-04 | 3E-04 | 0.002 | 0.003 | 0 | 1E-03 | 0.002 | 0.005 | 0.008 | 6E-05 | 4E-04 | 0.002 | 0.004 | 0.005 | 0 | 4E-04 | 9E-05 | 0.005 | 4E-04 | 7E-04 | 0 | 0.002 | 0.005 | 5E-04 | 0 | 8E-04 | 0.005 | 0.001 |
| Saccharomycetales | 0.002918 | 0.014 | 0.006 | 0.015 | 0.022 | 0.027 | 1E-03 | 2E-04 | 0.01 | 0.006 | 0.003 | 0.012 | 0.002 | 2E-04 | 0.002 | 0.006 | 0.008 | 0.003 | 3E-04 | 0.02 | 0.002 | 0.005 | 0.003 | 0.002 | 9E-04 | 1E-04 | 0.005 | 0.002 | 0.003 | 0.002 | 0.003 | 0.001 | 0.001 | 1E-04 | 0.001 | 0.015 | 0.007 |
| Filobasidiales | 0.002381 | 0.004 | 0.001 | 0.004 | 0 | 0.008 | 0.001 | 0.002 | 0.014 | 0.002 | 0.009 | 0.003 | 0.004 | 5E-04 | 0.002 | 0.01 | 0.005 | 0.005 | 0.001 | 0.006 | 9E-04 | 0.002 | 0.002 | 0 | 0.002 | 4E-04 | 0.003 | 0.001 | 0.013 | 0.002 | 0.021 | 0.003 | 0.001 | 2E-04 | 0.003 | 0 | 0 |
| Dothideales | 0.002198 | 0.005 | 0.004 | 0.003 | 0 | 0.004 | 0.006 | 0 | 0.018 | 4E-04 | 0.042 | 4E-04 | 8E-04 | 0 | 6E-04 | 0.007 | 0.017 | 0.004 | 0 | 0.002 | 0.004 | 0.001 | 0.007 | 0.003 | 0 | 0 | 2E-04 | 0.001 | 9E-04 | 8E-04 | 2E-04 | 3E-04 | 1E-04 | 0.002 | 0 | 0 | 8E-04 |
| Pezizales | 0.00196 | 2E-04 | 0.07 | 5E-04 | 0.002 | 0.014 | 0.024 | 0 | 0.011 | 0 | 0 | 7E-05 | 0 | 0 | 4E-04 | 1E-04 | 0.002 | 9E-04 | 0 | 8E-05 | 0.005 | 0 | 9E-04 | 0.004 | 0.001 | 0 | 2E-04 | 3E-04 | 0 | 0 | 0.001 | 2E-04 | 6E-04 | 0 | 0 | 0.005 | 0 |
| Trichosphaeriales | 0.001441 | 0.021 | 9E-05 | 0.001 | 0 | 0 | 0 | 0.002 | 0.002 | 0 | 0.012 | 0.009 | 0 | 0 | 7E-05 | 0.002 | 5E-04 | 0 | 0 | 0 | 0 | 5E-04 | 2E-04 | 0 | 0 | 0 | 0.006 | 0 | 6E-04 | 3E-04 | 0.009 | 8E-04 | 0 | 0 | 0.004 | 0.004 | 0 |
| Agaricales | 0.001351 | 0.003 | 0.004 | 8E-05 | 0.002 | 7E-04 | 7E-05 | 2E-04 | 7E-05 | 4E-04 | 0.007 | 0.001 | 0 | 2E-04 | 0.001 | 0 | 0.005 | 0.002 | 9E-04 | 0.001 | 5E-04 | 7E-04 | 2E-04 | 9E-04 | 0 | 0 | 5E-04 | 8E-04 | 1E-04 | 1E-04 | 0.004 | 1E-04 | 9E-04 | 7E-04 | 0.002 | 0.004 | 0.019 |
| Tremellales | 0.00098 | 0.002 | 0.002 | 0.004 | 7E-04 | 0.001 | 9E-04 | 0 | 0.01 | 6E-04 | 0.006 | 0.001 | 0.001 | 0.001 | 1E-03 | 0.002 | 0.003 | 0.001 | 0 | 5E-04 | 0 | 8E-04 | 7E-04 | 1E-04 | 7E-04 | 4E-04 | 0.001 | 2E-04 | 0.009 | 0.002 | 5E-04 | 0 | 0.002 | 3E-04 | 0.005 | 0.003 | 0.001 |
| Sordariales | 0.000861 | 0.002 | 0 | 8E-04 | 0.037 | 0 | 0 | 0 | 1E-04 | 2E-04 | 0.002 | 0 | 0 | 4E-04 | 0 | 0 | 0.003 | 9E-04 | 0 | 1E-04 | 0 | 0 | 0.005 | 0 | 0 | 2E-04 | 0 | 2E-04 | 5E-04 | 0 | 0 | 9E-04 | 0 | 0 | 0 | 0 | 4E-04 |
| Glomerellales | 0.000847 | 5E-04 | 0 | 0 | 0 | 0 | 0 | 0 | 0 | 4E-04 | 2E-04 | 9E-04 | 9E-05 | 0 | 4E-04 | 0 | 8E-04 | 2E-04 | 0 | 2E-04 | 0 | 0 | 0 | 0 | 0 | 7E-05 | 1E-04 | 0 | 0 | 0 | 0 | 6E-04 | 4E-04 | 0.006 | 0 | 0.003 | 0 |
| Sporidiobolales | 0.000797 | 4E-04 | 0 | 0 | 2E-04 | 0.002 | 0 | 0.002 | 0.009 | 1E-04 | 0.002 | 0.003 | 8E-05 | 2E-04 | 8E-04 | 0.002 | 0.004 | 0.002 | 0 | 0 | 0 | 8E-05 | 1E-04 | 0.001 | 0 | 1E-04 | 0.002 | 0 | 0 | 0.002 | 0.003 | 1E-03 | 0.001 | 0.004 | 0 | 0.006 | 0 |
| Polyporales | 0.000747 | 0.002 | 0 | 0 | 0 | 6E-04 | 0 | 0.003 | 0 | 3E-05 | 8E-04 | 7E-04 | 8E-05 | 1E-04 | 0 | 1E-04 | 6E-04 | 5E-04 | 4E-04 | 0.001 | 0.001 | 0 | 9E-04 | 0.002 | 0 | 0 | 0 | 2E-04 | 0 | 2E-04 | 0.002 | 0 | 1E-03 | 0 | 0 | 0.014 | 0 |
| Chaetothyriales | 0.000667 | 5E-04 | 0 | 0 | 0.003 | 0.002 | 0 | 0 | 0 | 4E-04 | 0.003 | 9E-04 | 9E-04 | 4E-05 | 0 | 0.001 | 0.002 | 0.001 | 0 | 0.002 | 0 | 2E-04 | 0 | 0.001 | 0 | 0 | 0.001 | 3E-04 | 0 | 0 | 1E-03 | 7E-04 | 0 | 0 | 0 | 0 | 0 |
| Wallemiales | 0.00061 | 0.002 | 0 | 4E-05 | 0 | 0 | 0 | 0.001 | 3E-04 | 9E-05 | 0.001 | 0.001 | 7E-04 | 5E-04 | 0.002 | 3E-04 | 0.002 | 5E-04 | 5E-04 | 0.001 | 6E-04 | 1E-03 | 0.004 | 0 | 8E-04 | 1E-04 | 0.001 | 4E-04 | 0.003 | 2E-04 | 0.012 | 3E-04 | 0.001 | 0.002 | 0.005 | 0.001 | 0 |
| Xylariales | 0.000581 | 0.002 | 0 | 3E-04 | 0 | 3E-04 | 0 | 2E-04 | 5E-04 | 1E-04 | 0.006 | 7E-04 | 0 | 0 | 4E-04 | 0 | 0.012 | 9E-04 | 0 | 0 | 0 | 5E-04 | 0 | 0 | 0 | 0 | 2E-04 | 6E-04 | 0.008 | 8E-05 | 9E-04 | 3E-04 | 5E-04 | 0 | 0 | 0 | 6E-04 |
| Erysiphales | 0.000484 | 8E-04 | 0 | 6E-05 | 0 | 0 | 0 | 0 | 0 | 4E-04 | 1E-03 | 1E-04 | 9E-05 | 1E-04 | 0 | 0.002 | 0 | 0.009 | 0 | 0 | 1E-03 | 0 | 6E-04 | 0 | 0 | 0 | 5E-04 | 0 | 6E-04 | 3E-05 | 0 | 0 | 0 | 0 | 0 | 0 | 0 |
| Entomophthorales | 0.000473 | 0 | 0 | 0 | 0 | 0 | 0 | 0 | 0.015 | 0 | 8E-05 | 0 | 0.017 | 0 | 0 | 0 | 0 | 0.004 | 0 | 0 | 0 | 0 | 0 | 0 | 0 | 0 | 4E-04 | 0 | 1E-04 | 0 | 0 | 0 | 0 | 0 | 0 | 0 | 0 |
| Botryosphaeriales | 0.000462 | 0 | 0 | 9E-05 | 0 | 0 | 0 | 0 | 0 | 0 | 3E-04 | 0 | 0 | 0 | 0 | 0 | 0.002 | 0 | 0 | 0 | 0.012 | 0 | 0 | 0 | 0 | 0 | 0 | 4E-04 | 0.001 | 0 | 0 | 0 | 0 | 0 | 0 | 0 | 0 |
| Cystofilobasidiales | 0.000455 | 0.003 | 0 | 0 | 0 | 0 | 0 | 0 | 0 | 0 | 0.001 | 3E-05 | 0 | 1E-04 | 3E-04 | 2E-04 | 1E-03 | 0.005 | 5E-04 | 4E-04 | 6E-04 | 6E-04 | 0 | 8E-04 | 0 | 0 | 0 | 0 | 0 | 0 | 0.005 | 0 | 2E-04 | 0.007 | 5E-04 | 0.003 | 0 |
| Ascomycota unidentified | 0.000359 | 1E-03 | 0 | 9E-05 | 7E-04 | 0 | 8E-05 | 0 | 0 | 0 | 9E-04 | 0.001 | 4E-05 | 0 | 9E-05 | 2E-04 | 0 | 0 | 0 | 5E-04 | 0.002 | 0 | 0 | 0 | 0 | 0 | 4E-04 | 0 | 2E-04 | 0 | 0.003 | 0 | 0 | 3E-05 | 0 | 0 | 0 |
| Dothideomycetes unidentified | 0.00028 | 0 | 0 | 0 | 0 | 0 | 0 | 0 | 0 | 0 | 7E-05 | 0 | 0 | 0 | 2E-04 | 0 | 0 | 0 | 0 | 0 | 0 | 0 | 0 | 0 | 0 | 0 | 0 | 0 | 0 | 4E-04 | 0 | 0 | 0 | 0 | 0 | 0.004 | 0 |
| Erythrobasidiales | 0.000239 | 0 | 0 | 0 | 0 | 0 | 0 | 0.001 | 0 | 7E-05 | 2E-04 | 0.001 | 9E-04 | 4E-05 | 3E-04 | 7E-04 | 0.002 | 0 | 7E-04 | 1E-04 | 0 | 2E-04 | 3E-04 | 0 | 0 | 0 | 5E-04 | 0 | 0 | 1E-04 | 0.005 | 5E-04 | 0 | 0 | 0 | 0 | 0 |
| Trichosporonales | 0.000195 | 0 | 7E-04 | 0 | 1E-04 | 4E-04 | 0 | 0 | 0 | 6E-05 | 0 | 5E-04 | 0 | 0 | 2E-04 | 0 | 0 | 3E-05 | 6E-04 | 3E-04 | 0 | 0.003 | 0.001 | 0 | 7E-04 | 0 | 0.001 | 0 | 6E-04 | 7E-04 | 2E-04 | 0 | 7E-04 | 0 | 9E-04 | 0 | 4E-04 |
| Cystobasidiales | 0.000177 | 0 | 0 | 0 | 0 | 4E-04 | 0 | 0 | 0.007 | 2E-04 | 0 | 2E-04 | 0 | 4E-05 | 2E-04 | 0.001 | 0 | 0 | 0 | 0 | 4E-04 | 0 | 3E-04 | 0 | 1E-04 | 0 | 3E-04 | 0 | 0 | 0 | 0.002 | 5E-04 | 4E-04 | 0 | 3E-04 | 0 | 0 |
| Helotiales | 0.000176 | 3E-04 | 0 | 0 | 0 | 0 | 0 | 0 | 0 | 4E-05 | 4E-04 | 4E-04 | 1E-04 | 0 | 4E-05 | 0 | 3E-04 | 0 | 0 | 0 | 0 | 0 | 0 | 0 | 2E-04 | 0 | 0 | 0 | 9E-05 | 0 | 7E-04 | 0.003 | 0 | 0 | 1E-04 | 0 | 0 |
| Tubeufiales | 0.000169 | 6E-04 | 0 | 0 | 0 | 0 | 0 | 0 | 5E-04 | 0 | 4E-04 | 1E-03 | 0.002 | 0 | 0 | 1E-04 | 0 | 1E-04 | 0 | 3E-04 | 0 | 0 | 0 | 0 | 0 | 0 | 0 | 0 | 0 | 0 | 0.002 | 7E-05 | 0 | 0 | 0 | 0 | 0 |
| Cantharellales | 0.000163 | 4E-04 | 0 | 0 | 0 | 0 | 0 | 0 | 0 | 0 | 0 | 1E-04 | 0 | 0 | 0 | 0 | 0 | 0 | 0 | 0 | 0 | 0 | 0 | 0 | 0 | 0 | 1E-04 | 0 | 1E-04 | 0 | 0 | 0.003 | 0 | 0 | 2E-04 | 0.001 | 0.002 |
| Ustilaginales | 0.000129 | 4E-04 | 3E-04 | 0 | 0 | 0 | 0 | 3E-04 | 0 | 4E-05 | 9E-04 | 5E-04 | 0 | 0 | 4E-05 | 1E-04 | 0.001 | 2E-04 | 0 | 2E-04 | 0 | 0 | 0 | 6E-04 | 3E-04 | 0 | 0 | 8E-05 | 2E-04 | 0 | 0.001 | 0 | 0 | 0 | 0 | 0 | 0 |
| Mortierellales | 0.000126 | 4E-04 | 0 | 0 | 0 | 0 | 0 | 0 | 0 | 0 | 0 | 1E-03 | 0 | 0 | 0 | 0 | 0 | 0 | 0 | 0 | 0 | 0 | 0 | 0 | 0 | 0 | 6E-05 | 0 | 0 | 2E-04 | 0 | 0.002 | 0 | 0.003 | 0 | 0 | 0.002 |
| Thelebolales | 0.000126 | 0 | 0 | 0 | 0 | 0 | 0 | 0 | 0 | 0 | 0 | 0 | 0 | 0 | 0 | 0 | 2E-04 | 0 | 0 | 0 | 0 | 0 | 0 | 0 | 0 | 0 | 0 | 0 | 2E-04 | 0 | 0 | 0.003 | 0 | 0 | 0 | 0 | 0 |
| Agaricostilbales | 0.000121 | 0 | 0 | 0 | 0 | 0.002 | 0 | 0 | 0 | 0 | 0.001 | 1E-04 | 0 | 7E-05 | 0 | 8E-05 | 3E-04 | 5E-04 | 0 | 0 | 0 | 0 | 0 | 0 | 0 | 0 | 0 | 0 | 0 | 0 | 0 | 0 | 0 | 0.003 | 0 | 0 | 0 |
| Cystobasidiomycetes unidentified | 0.000121 | 0 | 0 | 0.002 | 3E-04 | 0 | 4E-04 | 0 | 0 | 0 | 0 | 3E-04 | 5E-04 | 0 | 0 | 4E-05 | 0.002 | 0 | 0 | 4E-04 | 0 | 0 | 0 | 0 | 0 | 0 | 7E-04 | 0 | 0.001 | 0 | 9E-04 | 0 | 4E-04 | 0 | 0 | 0 | 0 |
| Basidiomycota unidentified | 0.000117 | 1E-03 | 0 | 0 | 0 | 0 | 0 | 0 | 0 | 0 | 0.001 | 0.001 | 9E-05 | 0 | 0 | 0 | 4E-04 | 0 | 0 | 6E-04 | 9E-04 | 0 | 0 | 0 | 2E-04 | 0 | 0.001 | 0 | 0 | 0 | 0 | 6E-04 | 0 | 0 | 0 | 0 | 0 |
| Microbotryomycetes unidentified | 0.00016 | 0 | 4E-05 | 0 | 9E-04 | 0 | 0 | 0 | 0 | 1E-04 | 6E-04 | 5E-04 | 0 | 0 | 2E-04 | 0 | 7E-04 | 0 | 0.007 | 8E-05 | 0 | 4E-04 | 0 | 0 | 0 | 0 | 2E-04 | 0 | 2E-04 | 2E-04 | 0 | 3E-04 | 2E-04 | 6E-04 | 0 | 0 | 0 |
| Unassigned | 9.63E-05 | 0 | 0 | 0 | 0 | 3E-04 | 2E-04 | 0 | 0 | 0 | 2E-04 | 7E-04 | 6E-05 | 3E-04 | 7E-04 | 0 | 6E-04 | 2E-04 | 0 | 0 | 0 | 0 | 3E-04 | 0 | 3E-04 | 4E-04 | 6E-04 | 7E-04 | 0 | 2E-04 | 0 | 0 | 6E-04 | 0 | 2E-04 | 0 | 0 |
| Russulales | 6.63E-05 | 0 | 0 | 0 | 0 | 0 | 0 | 0 | 0 | 0 | 0 | 0 | 1E-04 | 0 | 9E-05 | 3E-04 | 0 | 5E-04 | 0 | 8E-04 | 0 | 0 | 0 | 0 | 0 | 0 | 0 | 5E-04 | 0 | 0 | 0 | 0 | 0 | 0 | 0 | 0.001 | 0 |
| Agaricomycetes unidentified | 6.12E-05 | 0 | 0 | 0 | 3E-04 | 0 | 9E-04 | 0 | 0 | 0 | 1E-04 | 0 | 0 | 0 | 0 | 0 | 0.003 | 0 | 0 | 0 | 0 | 0 | 0 | 0 | 0 | 0 | 0 | 0 | 0 | 0 | 0 | 4E-04 | 0 | 0 | 0 | 0 | 0 |
| Rozellomycota unidentified | 5.9E-05 | 0 | 0 | 0 | 0 | 0 | 0.001 | 0 | 0 | 0 | 0 | 1E-04 | 0 | 0 | 0 | 0 | 0 | 0 | 0 | 0 | 0 | 0 | 0 | 0 | 0 | 0 | 0 | 0 | 0 | 0 | 0 | 4E-04 | 0 | 0 | 0 | 0 | 8E-04 |
| Diaporthales | 5.01E-05 | 0 | 0 | 3E-04 | 0 | 0 | 0 | 0 | 0 | 0 | 1E-04 | 0 | 0 | 0 | 0 | 8E-05 | 0.003 | 0 | 0 | 0 | 0 | 0 | 9E-05 | 0 | 0 | 0 | 6E-05 | 0 | 0 | 0 | 0 | 0 | 0 | 0 | 0 | 0 | 0 |
| Sordariomycetes unidentified | 4.87E-05 | 0 | 0 | 0 | 0 | 0 | 0 | 0 | 0 | 0 | 7E-05 | 7E-04 | 0 | 0 | 0 | 1E-04 | 5E-04 | 0 | 0 | 0 | 0 | 0 | 8E-04 | 0 | 3E-04 | 0 | 0 | 0 | 0 | 0 | 0 | 3E-04 | 0 | 0 | 0 | 0 | 0 |
| Lichenostigmatales | 4.84E-05 | 0 | 0 | 0 | 0 | 0 | 0 | 0 | 0 | 0 | 2E-04 | 0 | 0 | 0 | 0 | 0 | 0 | 0 | 0 | 2E-04 | 0 | 0 | 0 | 0 | 0 | 0 | 0 | 0 | 0 | 0 | 0.003 | 0 | 0 | 0 | 0 | 0 | 0 |
| Tremellomycetes unidentified | 4.19E-05 | 0 | 0 | 0.001 | 0 | 0 | 0 | 0 | 4E-04 | 0 | 0 | 0 | 0 | 0 | 0 | 2E-04 | 0 | 0 | 0 | 0 | 0 | 0 | 0 | 0 | 0 | 0 | 0 | 0 | 0 | 0 | 0 | 8E-04 | 0 | 0 | 0 | 0 | 0 |
| Leucosporidiales | 4.04E-05 | 0 | 0 | 0 | 0 | 0 | 0 | 0 | 0 | 0 | 0 | 0 | 0 | 0 | 0 | 0 | 0 | 0 | 0 | 0 | 0 | 0 | 0 | 0 | 0 | 0 | 0 | 0 | 0 | 0 | 0 | 0 | 0 | 0 | 0 | 0 | 0 |
| Auriculariales | 3.71E-05 | 8E-04 | 0 | 0 | 0 | 0 | 0 | 0 | 0 | 0 | 0 | 0 | 0 | 0 | 0 | 0 | 0 | 0 | 0 | 0 | 0 | 0 | 0 | 0 | 0 | 0 | 0 | 0 | 0 | 0 | 0 | 3E-04 | 0 | 0 | 0 | 0 | 0 |

| | | | | | | | | | | | | | | | | | | | | | | | | | | | | | | | | | | | | | |
|---|---|---|---|---|---|---|---|---|---|---|---|---|---|---|---|---|---|---|---|---|---|---|---|---|---|---|---|---|---|---|---|---|---|---|---|---|---|
| Cryptomycota unidentified | 3.62E-05 | 0 | 0 | 0 | 0 | 0 | 0 | 0 | 0 | 0 | 0 | 0 | 5E-04 | 0 | 0 | 0 | 0 | 0 | 0 | 0 | 0 | 0 | 0 | 0 | 0 | 0 | 0 | 0 | 0 | 0 | 0 | 0 | 0 | 0 | 0 | 0 | 0 |
| Microascales | 3.51E-05 | 0 | 0 | 0 | 0 | 0 | 0 | 0 | 0 | 6E-05 | 8E-05 | 0 | 0 | 0 | 0 | 0 | 0 | 1E-04 | 0 | 0 | 0 | 0 | 0 | 0 | 3E-05 | 0 | 0 | 0 | 0 | 0 | 0 | 0 | 0 | 0.002 | 0 | 0 | 0 |
| Pezizomycetes unidentified | 3.42E-05 | 0 | 0 | 0 | 0 | 0 | 0 | 0 | 0 | 0 | 0 | 0 | 0 | 0 | 0 | 0 | 0 | 0 | 0 | 0 | 0 | 0 | 0 | 0 | 0 | 0 | 0 | 0 | 0 | 0 | 0 | 0 | 0 | 0 | 0 | 0 | 0 |
| Onygenales | 3.12E-05 | 0 | 0 | 0 | 0 | 0 | 0 | 0 | 0 | 0 | 0 | 0 | 4E-05 | 0 | 0 | 0 | 0 | 0 | 0 | 2E-04 | 0 | 0 | 0 | 0 | 0 | 0 | 0 | 0 | 0 | 0 | 0 | 0 | 0 | 0 | 0 | 0 | 0 |
| Mucoromycota unidentified | 2.45E-05 | 0 | 0 | 0 | 0 | 0 | 0 | 0 | 0 | 0 | 0 | 0 | 0 | 0 | 0 | 0 | 0 | 0 | 0 | 0 | 0 | 0 | 0 | 0 | 0 | 0 | 0 | 0 | 0 | 0 | 0 | 0 | 0 | 0 | 0 | 0 | 0 |
| Golubeviales | 2.35E-05 | 0 | 0 | 0 | 0 | 0 | 0 | 0 | 0 | 7E-05 | 0 | 0 | 0 | 1E-04 | 9E-05 | 0 | 0 | 0 | 0 | 0 | 0 | 0.001 | 0 | 1E-04 | 0 | 0 | 0 | 0 | 0 | 0 | 0 | 0 | 0 | 0 | 0 | 0 | 0 |
| Corticiales | 2.33E-05 | 0 | 0 | 0 | 0 | 0 | 0 | 0 | 0 | 0 | 0 | 0 | 0 | 0 | 0 | 0 | 0.002 | 0 | 0 | 0 | 0 | 0 | 0 | 0 | 0 | 0 | 0 | 0 | 0 | 0 | 0 | 0 | 0 | 0 | 0 | 0 | 0 |
| Mycosphaerellales | 2.31E-05 | 0 | 0 | 0 | 0 | 0 | 0 | 0 | 0 | 0 | 0 | 0 | 0 | 0 | 0 | 0 | 0 | 0 | 0 | 0 | 0 | 0 | 0 | 0 | 0 | 0 | 0 | 0 | 0 | 0 | 0 | 0 | 0 | 0 | 0 | 0 | 0 |
| Pezizomycotina unidentified | 2.07E-05 | 0 | 0 | 0 | 0 | 0 | 0 | 0 | 0 | 0 | 0 | 0 | 0 | 0 | 0 | 0 | 0 | 0.002 | 0 | 0 | 0 | 0 | 0 | 0 | 0 | 0 | 0 | 0 | 0 | 0 | 0 | 0 | 0 | 0 | 0 | 0 | 0 |
| Myrmecridiales | 1.96E-05 | 0 | 0 | 0 | 0 | 0 | 0 | 0 | 6E-04 | 0 | 0 | 4E-04 | 0 | 0 | 2E-04 | 1E-04 | 0 | 0 | 0 | 0 | 0 | 0 | 0 | 0 | 0 | 0 | 0 | 0 | 0 | 0 | 0 | 0 | 0 | 0 | 0 | 0 | 0 |
| Venturiales | 1.78E-05 | 0 | 2E-04 | 0 | 0 | 0 | 0 | 0 | 0 | 7E-05 | 2E-04 | 0 | 0 | 0 | 0 | 0 | 5E-04 | 0 | 0 | 0 | 0 | 0 | 0 | 0 | 0 | 0 | 0 | 0 | 0 | 0 | 0 | 0 | 0 | 0 | 0 | 0 | 0 |
| Myriangiales | 1.76E-05 | 0 | 0 | 0 | 0 | 0 | 0 | 0 | 0 | 0 | 2E-04 | 0 | 0 | 0 | 0 | 0 | 0 | 0 | 0 | 0 | 0 | 0 | 0 | 0 | 0 | 0 | 0 | 0 | 0 | 0 | 0 | 0 | 0 | 0 | 0 | 0 | 0 |
| Exobasidiales | 1.43E-05 | 0 | 0 | 7E-04 | 0 | 0 | 0 | 1E-04 | 0 | 0 | 0 | 0 | 0 | 9E-05 | 0 | 0 | 0 | 1E-04 | 0 | 0 | 0 | 0 | 0 | 0 | 0 | 0 | 0 | 6E-05 | 0 | 0 | 0 | 0 | 0 | 0 | 0 | 0 | 0 |
| Pyxidiophorales | 1.19E-05 | 0 | 6E-04 | 0 | 0 | 0 | 0 | 0 | 0 | 0 | 8E-05 | 0 | 0 | 0 | 0 | 0 | 0 | 0 | 0 | 0 | 3E-04 | 0 | 0 | 0 | 0 | 0 | 0 | 0 | 0 | 0 | 0 | 0 | 0 | 0 | 0 | 0 | 0 |
| Orbiliales | 8.31E-06 | 0 | 0 | 0 | 0 | 0 | 0 | 0 | 0 | 0 | 0 | 0 | 0 | 0 | 0 | 0 | 0 | 0 | 0 | 0 | 0 | 0 | 0 | 0 | 0 | 0 | 0 | 0 | 0 | 0 | 0 | 0 | 0 | 0 | 0 | 0 | 0 |
| Lulworthiales | 7.48E-06 | 0 | 0 | 0 | 0 | 0 | 0 | 0 | 0 | 0 | 0 | 0 | 0 | 0 | 0 | 0 | 0 | 0 | 0 | 0 | 0 | 0 | 0 | 0 | 0 | 0 | 0 | 0 | 0 | 0 | 0 | 0 | 0 | 0 | 0 | 0 | 0 |
| Mucorales | 7.19E-06 | 0 | 0 | 0 | 0 | 0 | 0 | 0 | 0 | 0 | 0 | 0 | 0 | 4E-05 | 0 | 0 | 0 | 5E-04 | 0 | 0 | 0 | 0 | 0 | 0 | 0 | 0 | 0 | 0 | 0 | 0 | 0 | 0 | 0 | 0 | 0 | 0 | 0 |
| Taphrinales | 6.3E-06 | 0 | 0 | 0 | 0 | 0 | 0 | 0 | 0 | 0 | 0 | 0 | 0 | 0 | 0 | 0 | 0 | 0 | 0 | 5E-04 | 0 | 0 | 0 | 0 | 0 | 0 | 0 | 0 | 0 | 0 | 0 | 0 | 0 | 0 | 0 | 0 | 0 |
| Ascomycota GS35 | 6.21E-06 | 0 | 0 | 0 | 0 | 0 | 0 | 0 | 0 | 0 | 0 | 0 | 0 | 0 | 0 | 0 | 0 | 0 | 0 | 0 | 0 | 0 | 0 | 0 | 0 | 0 | 0 | 0 | 0 | 0 | 0 | 5E-04 | 0 | 0 | 0 | 0 | 0 |
| Atheliales | 5.82E-06 | 0 | 0 | 0 | 0 | 0 | 0 | 0 | 0 | 0 | 0 | 0 | 0 | 0 | 0 | 0 | 0 | 0 | 0 | 0 | 0 | 0 | 0 | 0 | 0 | 0 | 0 | 0 | 0 | 0 | 0 | 0 | 0 | 0 | 0 | 0 | 0 |
| Thelephorales | 5.73E-06 | 0 | 0 | 0 | 0 | 0 | 0 | 0 | 0 | 0 | 0 | 0 | 0 | 0 | 0 | 0 | 0 | 0 | 0 | 0 | 0 | 0 | 0 | 0 | 0 | 0 | 0 | 0 | 0 | 0 | 0 | 4E-04 | 0 | 0 | 0 | 0 | 0 |
| Archaeorhizomycetales | 5.5E-06 | 0 | 0 | 0 | 0 | 0 | 0 | 0 | 0 | 0 | 0 | 0 | 0 | 0 | 0 | 0 | 0 | 0 | 0 | 0 | 4E-04 | 0 | 0 | 0 | 0 | 0 | 0 | 0 | 0 | 0 | 0 | 7E-05 | 0 | 0 | 0 | 0 | 0 |
| Gloeophyllales | 5.11E-06 | 0 | 0 | 0 | 0 | 0 | 0 | 0 | 0 | 0 | 0 | 4E-04 | 0 | 0 | 0 | 0 | 0 | 0 | 0 | 0 | 0 | 0 | 0 | 0 | 0 | 0 | 0 | 0 | 0 | 0 | 0 | 0 | 0 | 0 | 0 | 0 | 0 |
| Leotiomycetes unidentified | 4.57E-06 | 0 | 0 | 0 | 0 | 0 | 0 | 0 | 0 | 0 | 0 | 0 | 0 | 0 | 0 | 0 | 0 | 0 | 0 | 0 | 0 | 0 | 0 | 0 | 0 | 0 | 0 | 0 | 0 | 0 | 0 | 0 | 0 | 0 | 0 | 0 | 0 |
| Hymenochaetales | 4.01E-06 | 0 | 0 | 0 | 0 | 0 | 0 | 0 | 0 | 7E-05 | 0 | 0 | 0 | 0 | 0 | 0 | 0 | 2E-04 | 0 | 0 | 0 | 0 | 0 | 0 | 0 | 0 | 0 | 0 | 0 | 0 | 0 | 0 | 0 | 0 | 0 | 0 | 0 |
| Eurotiomycetes unidentified | 3.87E-06 | 0 | 0 | 0 | 0 | 0 | 0 | 0 | 0 | 0 | 3E-04 | 0 | 0 | 0 | 0 | 0 | 0 | 0 | 0 | 0 | 0 | 0 | 0 | 0 | 0 | 0 | 0 | 0 | 0 | 0 | 0 | 0 | 0 | 0 | 0 | 0 | 0 |
| Rhizophlyctidales | 3.54E-06 | 0 | 0 | 0 | 0 | 0 | 0 | 0 | 0 | 0 | 0 | 0 | 0 | 0 | 0 | 0 | 0 | 0 | 0 | 0 | 0 | 0 | 0 | 0 | 3E-04 | 0 | 0 | 0 | 0 | 0 | 0 | 0 | 0 | 0 | 0 | 0 | 0 |
| Melanosporales | 2.87E-06 | 0 | 0 | 0 | 0 | 0 | 0 | 0 | 1E-04 | 1E-04 | 0 | 0 | 0 | 0 | 0 | 0 | 0 | 0 | 0 | 0 | 0 | 0 | 0 | 0 | 0 | 0 | 0 | 0 | 0 | 0 | 0 | 0 | 0 | 0 | 0 | 0 | 0 |
| Kriegeriales | 2.86E-06 | 0 | 0 | 0 | 0 | 0 | 0 | 0 | 0 | 0 | 0 | 0 | 0 | 0 | 0 | 0 | 0 | 0 | 0 | 0 | 0 | 0 | 0 | 0 | 0 | 0 | 0 | 0 | 0 | 0 | 0 | 2E-04 | 0 | 0 | 0 | 0 | 0 |
| Chytridiomycota unidentified | 2.86E-06 | 0 | 0 | 0 | 0 | 0 | 0 | 0 | 0 | 0 | 0 | 0 | 0 | 0 | 0 | 0 | 0 | 0 | 0 | 0 | 0 | 0 | 0 | 0 | 0 | 0 | 0 | 0 | 0 | 0 | 0 | 2E-04 | 0 | 0 | 0 | 0 | 0 |
| Rozellomycotina GS11 | 2.36E-06 | 0 | 0 | 0 | 0 | 0 | 0 | 0 | 0 | 0 | 2E-04 | 0 | 0 | 0 | 0 | 0 | 0 | 0 | 0 | 0 | 0 | 0 | 0 | 0 | 0 | 0 | 0 | 0 | 0 | 0 | 0 | 0 | 0 | 0 | 0 | 0 | 0 |
| Lecanorales | 1.65E-06 | 0 | 0 | 0 | 0 | 0 | 0 | 0 | 0 | 0 | 0 | 0 | 0 | 0 | 0 | 0 | 0 | 0 | 0 | 0 | 0 | 0 | 0 | 0 | 0 | 0 | 0 | 0 | 0 | 0 | 0 | 0 | 0 | 0 | 0 | 0 | 0 |
| Glomeromycetes unidentified | 1.15E-06 | 0 | 0 | 0 | 0 | 0 | 0 | 0 | 0 | 0 | 0 | 0 | 0 | 0 | 0 | 0 | 0 | 0 | 0 | 0 | 0 | 0 | 9E-05 | 0 | 0 | 0 | 0 | 0 | 0 | 0 | 0 | 0 | 0 | 0 | 0 | 0 | 0 |
| Entylomatales | 1.09E-06 | 0 | 0 | 0 | 0 | 0 | 8E-05 | 0 | 0 | 0 | 0 | 0 | 0 | 0 | 0 | 0 | 0 | 0 | 0 | 0 | 0 | 0 | 0 | 0 | 0 | 0 | 0 | 0 | 0 | 0 | 0 | 0 | 0 | 0 | 0 | 0 | 0 |
| Tilletiales | 8.13E-07 | 0 | 0 | 0 | 0 | 0 | 0 | 0 | 0 | 0 | 0 | 0 | 0 | 0 | 0 | 0 | 0 | 0 | 0 | 0 | 0 | 0 | 0 | 0 | 0 | 0 | 0 | 0 | 0 | 0 | 0 | 0 | 6E-05 | 0 | 0 | 0 | 0 |
| Trechisporales | 4.29E-07 | 0 | 0 | 0 | 0 | 0 | 0 | 0 | 0 | 0 | 0 | 0 | 0 | 0 | 0 | 0 | 0 | 0 | 0 | 0 | 0 | 0 | 0 | 0 | 0 | 0 | 0 | 0 | 0 | 0 | 0 | 0 | 0 | 0 | 0 | 0 | 0 |

**Table S7 The information of isolated Talaromyces strains**

| Strain number | Closest strain | Accession number | Taxa | Sequence |
|---|---|---|---|---|
| XSF1 | Talaromyces funiculosus isolate S73-1 | MT078712 | Ascomycota; Eurotiomycetes; Eurotiales; Trichocomaceae; Talaromyces. | GGCGGGCCCACTGGGGCTCCCTGGTCGCCGGGGGACACCCGTCCCCGGGCCCGCGCCCGCCGAAGCGCTTCGTGAACCCTG<br>ATGAAGAAGGGCTGTCTGAGTACTATGAAAATTGTCAAAACTTTCAACAATGGATCTCTTGGTTCCGGCATCGATGAAGAA<br>CGCAGCGAAATGCGATAAGTAATGTGAATTGCAGAATTCCGTGAATCATCGAATCTTTGAACGCACATTGCGCCCCCTGGC<br>ATTCCGGGGGGCATGCCTGTCCGAGCGTCATTTCTGCCCTCAAGCACGGCTTGTGTGTTGGGTGTGGTCCCCCCGGGGACCT<br>GCCCGAAAGGCAGCGGCGACGTCCGTCTGGTCCTCGAGCGTATGGGGCTCTGTCACTCGCTCGGGAAGGACCTGCGGGGGT<br>TGGT |
| XSF5 | Talaromyces funiculosus isolate S73-1 | MT078712 | Ascomycota; Eurotiomycetes; Eurotiales; Trichocomaceae; Talaromyces. | TTGGCGGGCCCACTGGGGCTCCCTGGTCGCCGGGGGACACCCGTCCCCGGGCCCGCGCCCGCCGAAGCGCTTCGTGAACCC<br>TGATGAAGAAGGGCTGTCTGAGTACTATGAAAATTGTCAAAACTTTCAACAATGGATCTCTTGGTTCCGGCATCGATGAAG<br>AACGCAGCGAAATGCGATAAGTAATGTGAATTGCAAAATTCCGTGAATCATCGAATCTTTGAACGCACATTGCGCCCCCTG<br>GCATTCCGGGGGGCATGCCTGTCCGAGCGTCATTTCTGCCCTCAAGCACGGCTTGTGTGTTGGGTGTGGTCCCCCCGGGGA<br>CCTGCCCGAAAGGCAGCGGCGACGTCCGTCTGGTCCTCGAGCGTATGGGGCTCTGTCACTCGCTCGGGAAGGACCTGCGGG<br>GGTTG |
| XSF7 | Talaromyces funiculosus strain MSS612 | MK805486 | Ascomycota; Eurotiomycetes; Eurotiales; Trichocomaceae; Talaromyces | GCTTTGGCGGGCCCACCGGGGCCACCTGGTCGCCGGGGGACGTTCGTCCCCGGGCCCGCGCCCGCCGAAGCGCTCTGTGAA<br>CCCTGATGAAGATGGGCTGTCTGAGTACTATGAAAATTGTCAAAACTTTCAACAATGGATCTCTTGGTTCCGGCATCGATG<br>AAGAACGCAGCGAAATGCGATAAGTAATGTGAATTGCAGAATTCCGTGAATCATCGAATCTTTGAACGCACATTGCGCCCC<br>CTGGCATTCCGGGGGGCATGCCTGTCCGAGCGTCATTTCTGCCCTCAAGCACGGCTTGTGTGTTGGGTGCGGTCCCCCCGG<br>GGGCCTGCCCGAAAGGCAGCGGCGACGTCCGTCTGGTCCTCGAGCGTATGGGGCTTTGTCACTCGCTCGGGAAGGACTGGC<br>GGGGG |
| XSF10 | Talaromyces purpureogenus strain XD520 | MK805487 | Ascomycota; Eurotiomycetes; Eurotiales; Trichocomaceae; Talaromyces | TCGGCGGGCCCACCGGGGCCACCCGGTCGCCGGGGGACATCCGTCCCCGGGCCCGCGCCCGCCGAGGCGCTCTGTGAACC<br>CTGATGAAGATGGGCTGTCTGAGTGATATGAAAATTGTCAAAACTTTCAACAATGGATCTCTTGGTTCCGGCATCGATGAA<br>GAACGCAGCGAAATGCGATAAGTAATGTGAATTGCAGAATTCCGTGAATCATCGAATCTTTGAACGCACATTGCGCCCCCT<br>GGCATTCCGGGGGGCATGCCTGTCCGAGCGTCATTTCTGCCCTCAAGCACGGCTTGTGTGTTGGGTGTGGTCCCCCTGGGG<br>ACCTGCCCGAAAGGCAGCGGCGACGTCCGTCTGGTCCTCGAGCGTATGGGGCTCTGTCACTCGCTCGGGAAGGACCTGCGG<br>GGGTTG |
| XSF103 | Talaromyces radicus strain TBS21202 | MK805489 | Ascomycota; Eurotiomycetes; Eurotiales; Trichocomaceae;Talaromyces. | TCGGCGGGCCCACTGGGGCCTGGCCCCGGTCGCCGGGGGGCTTCTGCCCCCGGGCCCGCGCCCGCCGACGCACCCTAGAA<br>CCCTGCCTGAATAGTGAGTCTGAGTGAGATTTGAAATCATTAAAACTTTCAACAACGGATCTCTTGGTTCCGGCATCGATG<br>AAGAACGCAGCGAAATGCGATAAGTAATGTGAATTGCAGAATTCCGTGAATCATCGAATCTTTGAACGCACATTGCGCCCC<br>CTGGCATTCCGGGGGGCATGCCTGTCCGAGCGTCATTTCTGCCCTCCAGCACGGCTGGGTGTTGGGCGCTGTCCCCCCGGG<br>GACACGCCCCAAAAGCAGTGGCGGCGCCGCGTCGGGTCCTCGAGCGTATGGGGCTTTGTCACCCGCTCGGGAGGGACTCG<br>GTCGGCG |
